# Diorganotin(IV) Complexes of Organoselenolato Ligands with Pyrazole Moieties—Synthesis, Structure and Properties †

**DOI:** 10.3390/molecules30071648

**Published:** 2025-04-07

**Authors:** Melinda Tamas, Roxana A. Butuza, Monica Dan, Anca Silvestru

**Affiliations:** 1 Supramolecular Organic and Organometallic Chemistry Centre (CCSOOM), Chemistry Department, Faculty of Chemistry and Chemical Engineering, “Babeş-Bolyai” University, 400028 Cluj-Napoca, Romania; melinda.tamas@ubbcluj.ro (M.T.); roxana.popa@ubbcluj.ro (R.A.B.); 2 National Institute for Research and Development of Isotopic and Molecular Technologies, 400293 Cluj-Napoca, Romania; monica.dan@itim-cj.ro

**Keywords:** diorganotin(IV) complexes, pyrazole-based organoselenolato ligands, solution behaviour, single-crystal X-ray diffraction, thermal behaviour

## Abstract

Diorganotin(IV) compounds of types RR′Sn(SeCH_2_CH_2_pz)_2_ [R = R′ = ^n^Bu (**2**), Ph (**3**); R = 2-(Me_2_NCH_2_)C_6_H_4_, R′ = Me (**4**), ^n^Bu (**5**), Ph (**6**)], and RR′SnX(SeCH_2_CH_2_pz) [R = 2-(Me_2_NCH_2_)C_6_H_4_, R′ = ^n^Bu, X = Cl (**7**), R′ = Me, X = SCN (**9**)], as well as [2-(Me_2_NCH_2_)C_6_H_4_](Me)Sn(NCS)_2_ (**8**), and the tin(II) Sn(SeCH_2_CH_2_pz)_2_ (**10**) (pz = pyrazole), were prepared by salt metathesis reactions between the appropriate diorganotin(IV) dichloride or dipseudohalide and Na[SeCH_2_CH_2_pz], with the latter freshly prepared from (pzCH_2_CH_2_)_2_Se_2_ (**1**). The solution behaviour of these compounds was investigated by multinuclear NMR (^1^H, ^13^C, ^77^Se, ^119^Sn), and the NMR spectra showed the existence of the Se–Sn bonds in solution. Compounds **4** and **5** showed decomposition in a solution of chlorinated solvents with the formation of selenium bridged dimeric species of type {[2-(Me_2_NCH_2_)C_6_H_4_](R’)Se}_2_ [R′ = Me (**4-a**), ^n^Bu (**5-a**)], as the single-crystal X-ray diffraction studies revealed, in contrast with compound **9,** for which a monomeric structure was observed with the desired composition. The solid state structures of **4-a**, **5-a**, **8**, and **9** revealed N→Sn intramolecular coordination of the nitrogen atom in the pendant CH_2_NMe_2_ arm. The NMR spectra suggested such a coordination at room temperature only for compound **7**.

## 1. Introduction

The potential of the organotin and the organoselenium compounds in biology, catalysis, and materials science determined over the past decades a continuously increasing interest to develop new chemical species, with an appropriate combination of organic groups and ligands, which might improve thermal and hydrolytic stability and enhance specific properties. Organoselenium compounds showed significant antioxidant, anti-inflammatory, and antiproliferative activity [1,2,3], were used as catalysts in organic synthesis [4,5], and were successfully employed as starting materials for precursors for nanoparticles or thin films [6]. On the other hand, organotin species displayed antitumor activity against several types of cancer [7,8,9], are used in catalysis [10,11], and have proven to be valuable precursors for nanomaterials [12] as well. These facts have sparked interest in obtaining chemical species which contain both tin and selenium in the same molecule, thus promising enhanced potential both for biological applications and nanomaterials.

Tin(II) and tin(IV) complexes with Sn–Se bonds displayed structural features and properties which recommended them as valuable single-source precursors for binary SnSe or SnSe_2_ thin films and nanoparticles [13,14,15]. SnSe is a p-type semiconductor, with a direct band gap of 0.9 eV and an indirect band gap of 1.3 eV [16,17], while SnSe_2_ is an n-type semiconductor with an indirect band gap of 1.0 eV [18]. Both show excellent thermoelectric and optoelectronic properties based on their intrinsic anisotropy [19]. The large number of applications of tin chalcogenides for optoelectronic devices, gas sensors, or anode materials for Li/Na-ion batteries determined a significant interest to find new single-source precursors, which allow for better control upon the thermal behaviour, structure, and phase composition when compared with dual-source precursors [20].

Several tin(II) complexes, e.g., ^1^_∞_[Sn(SePh)_2_] [17], Sn[SeSi(SiMe_3_)_3_] [21], and [Sn(2-Sepy)_2_] [22], were observed to undergo a single-step thermal decomposition to yield SnSe, while the tin(IV) complexes lead to SnSe nanoparticles by pyrolysis, i.e., [Sn(SePh)_4_] [23], or decomposed to SnSe_2_, i.e., [Sn(2-Sepy)_4_] [22]. The tin(IV) complexes [SnCl_2_{(SeCH_2_CH_2_)_2_NMe}] and [Sn{(SeCH_2_CH_2_)_2_NMe}_2_] were used as single-source precursors for SnSe nanoparticles, either by microwave-assisted solvothermal processes or by thermolysis [19]. Dinuclear triorganotin(IV) compounds, with bulky organic groups attached to tin, e.g., (Ph_3_Sn)_2_Se [24,25] and diorganotin(IV) species, either dinuclear, e.g., [{(Me_2_Si)_2_CH}_2_Sn(μ-Se)]_2_ [26], or mononuclear derivatives of types R_2_Sn(SeAr)_2_ and R_2_SnCl(SeAr) (R = Me, Et, ^n^Bu, ^t^Bu; Ar = C_5_H_4_N, C_5_H_3_(3-Me)N, C_5_H_3_(5-Me)N, C_4_H(4,6-Me_2_)_2_N_2_), were also studied by thermal decomposition or pyrolysis to obtain SnSe or SnSe_2_ nanoparticles [27,28,29]. In addition, atmospheric pressure chemical vapour deposition (APCVD) or aerosol-assisted CVD (AACVD) were employed to obtain SnSe thin films and nanosheets from diorganotin bis(organoselenolates) [29,30,31].

In this context, there still is significant interest in designing new compounds with thermal stability and high volatility, and to choose the most suitable combinations of organic groups attached both to tin and selenium, which not only enable a neat thermal decomposition, but also allow us to obtain nanocrystals, nanosheets, or thin films of tin selenides with homogeneous structures and compositions. It was shown that the use of organic groups decorated with pendant arms bearing nitrogen donor atoms capable for intramolecular coordination has a significant influence not only on the structure of the organometallic species, either organotin or organoselenium, but, more important, on their reactivity and specific behaviour in the above-mentioned fields [32,33,34]. As a consequence, various organic groups capable of behaving as *C*,*N* (e.g., 2-(Me_2_NCH_2_)C_6_H_4_) or *N*,*C*,*N* (e.g., 2,6-(Me_2_NCH_2_)C_6_H_3_) chelating moieties were employed both in organotin [35,36,37,38] and organoselenium [39,40,41,42] chemistry.

Previously, we reported on the tin complexes Sn[SeC_6_H_4_(CH_2_NMe_2_)-2][N(SiMe_3_)_2_] and Sn(SePh)_2_[N(SiMe_3_)_2_] [43], obtained by the reactions of Sn[N(SiMe_3_)_2_]_2_ with the corresponding diorganodiselenide, as well as homo- and heteroleptic di- and triorganotin(IV) complexes with pseudohalido ligands, i.e., [2-(Me_2_NCH_2_)C_6_H_4_]RSn(NCE)_2_ [E = S, Se; R = ^n^Bu, Ph, 2-(Me_2_NCH_2_)C_6_H_4_] and [2-(Me_2_NCH_2_)C_6_H_4_]R_2_Sn(NCE) (E = S, Se, R = Me, Ph) [44], or dithiocarbamato ligands, i.e., RR′Sn(S_2_CNR″_2_)Cl [R = R′ = Me, ^n^Bu; R″ = Me, Et; R = 2-(Me_2_NCH_2_)C_6_H_4_, R′ = Me, R″ = Me, Et] and RR′Sn(S_2_CNR″_2_)(NCS) [R = R′ = Me, ^n^Bu, R″ = Me, Et; R = 2-(Me_2_NCH_2_)C_6_H_4_, R′ = Me, ^n^Bu, R″ = Me, Et [45], prepared by salt metathesis reactions. Recently, we reported on diorganotin(IV) compounds with organoselenolato ligands bearing alkyl groups with pyrazole moieties, namely R_2_Sn(SeCH_2_CH_2_pz)_2_ [R = Me, 2-(Me_2_NCH_2_)C_6_H_4_], and we investigated their antiproliferative activity against murine colon carcinoma C26 cells [46]. As a continuation of our research on this topic, the present work reports on the synthesis and structural characterization of new homo- and heteroleptic diorganotin(IV) bis(organoselenolates) with pyrazole containing organic groups, namely RR′Sn(SeCH_2_CH_2_pz)_2_ [R = R′ = ^n^Bu (**2**), Ph (**3**); R = 2-(Me_2_NCH_2_)C_6_H_4_, R′ = Me (**4**), ^n^Bu (**5**), Ph (**6**)], [2-(Me_2_NCH_2_)C_6_H_4_](^n^Bu)SnCl(CH_2_CH_2_Se) (**7**), [2-(Me_2_NCH_2_)C_6_H_4_](Me)Sn(NCS)_2_ (**8**), and [2-(Me_2_NCH_2_)C_6_H_4_](Me)Sn(NCS)(SeCH_2_CH_2_pz) (**9**), as well as the tin(II) complex Sn(SeCH_2_CH_2_pz)_2_ (**10**).

## 2. Results and Discussion

### 2.1. Synthesis

**Scheme 1:**
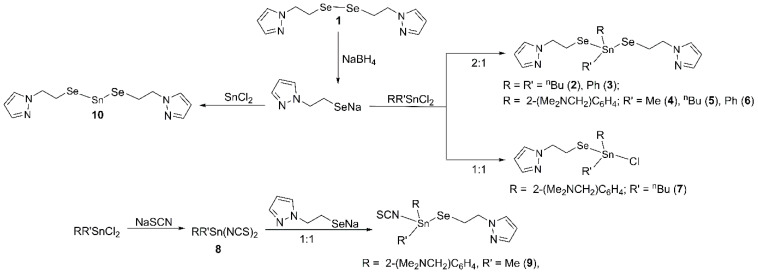
Synthesis of the tin complexes.

Diorganotin(IV) compounds of types RR′SnL_2_ [L = SeCH_2_CH_2_pz, R = R′ = ^n^Bu (**2**), Ph (**3**), R = 2-(Me_2_NCH_2_)C_6_H_4_, R′ = Me (**4**), ^n^Bu (**5**), Ph (**6**)], and RR′SnXL [L = SeCH_2_CH_2_pz, R = 2-(Me_2_NCH_2_)C_6_H_4_, R′ = ^n^Bu, X = Cl (**7**); R′ = Me, X = NCS (**9**)], as well as [2-(Me_2_NCH_2_)C_6_H_4_](Me)Sn(NCS)_2_ (**8**) and the tin(II) compound Sn(SeCH_2_CH_2_pz)_2_ (**10**), were prepared by salt metathesis reactions, as depicted in Scheme 1. The sodium organoselenolate was freshly prepared, starting from (pzCH_2_CH_2_)_2_Se_2_ (**1**). Compounds **4**, **8**, **9**, and **10** were isolated as solid species, while the other compounds were oils.

### 2.2. Spectroscopic Characterization

#### Solution Behaviour

All compounds were characterized by multinuclear NMR (^1^H, ^13^C{^1^H}, ^77^Se{^1^H}, and ^119^Sn{^1^H}, as appropriate) spectroscopy and mass spectrometry. The NMR spectra suggested in each case the presence of the desired species in solution.

Both the ^1^H and the ^13^C{^1^H} NMR spectra show the expected resonances for the organic groups attached to tin and to selenium, respectively (see the Supplementary Information (SI), Figures S1–S6). The ^1^H NMR spectra display for the CH_2_ groups in the pzCH_2_CH_2_Se^−^ ligands two multiplet resonances (see the Supplementary Information, Figures S1, S3, S5 and S6), with a splitting pattern determined by the non-equivalence of the two protons in each CH_2_ group.

The resonances of the protons and the carbon atoms in the proximity of tin or selenium are accompanied by ^117/119^Sn and ^77^Se satellites, respectively, and display characteristic coupling constants. The ^1^*J*_SnC_ coupling constant for compound **2**, which has two aliphatic carbons attached to tin, is given in Table 1. This value was used to calculate the C–Sn–C angle and to assign a distorted tetrahedral coordination geometry to tin in solution [47,48].

**Figure 1:**
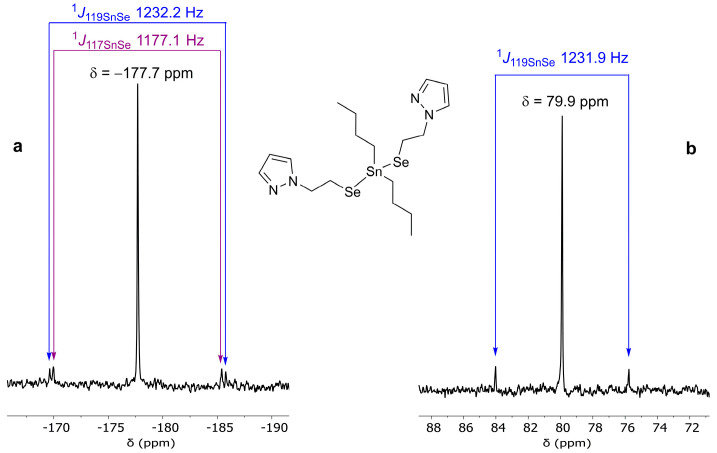
^77^Se{^1^H} (**a**) and ^119^Sn{^1^H} (**b**) resonances for compound **2**.

The ^119^Sn{^1^H} spectra of all compounds bearing the organoselenolato ligand show a singlet resonance in each case, accompanied by ^1^*J*_SeSn_ coupling constants. The ^119^Sn{^1^H} resonance of compound **9** has a different pattern (see the Supplementary Information, Figure S7), the splitting suggesting dipolar interactions with the two quadrupolar ^14^N nuclei and ^119^Sn-^14^N spin–spin coupling, as previously described by Wrackmeyer [49]. The ^77^Se{^1^H} spectra show a singlet resonance for each compound, accompanied by ^1^*J*_117SnSe_ and ^1^*J*_119SnSe_ coupling constants. The values for the ^1^*J*_SeSn_ coupling constants, along with the ^119^Sn and the ^77^Se chemical shifts for compounds **1**–**10**, and the starting diorganotin halides, are given in Table 2, while the ^119^Sn{^1^H} and the ^77^Se{^1^H} resonances for compound **2**, as an example, are displayed in Figure 1. Based on the NMR spectra, we can conclude that in each case, the organoselenolato ligand is attached to tin by selenium.

For the heteroleptic diorganotin(IV) compounds **4**–**9**, the presence of the 2-(Me_2_NCH_2_)C_6_H_4_ group might determine an increase in the coordination number at the tin atom, due to an intramolecular N→Sn coordination, in which the CH_2_N(CH_3_)_2_ pendant arm is involved. Such a secondary interaction should be observed in the ^1^H and the ^13^C NMR spectra by the no more equivalent C*H*_2_ protons and the two methyl groups in the CH_2_N(CH_3_)_2_ pendant arm. We noted this behaviour in solution, at room temperature, only for compound **7**, where the resonance for the C*H*_2_ protons appears as an AB spin system (δ_A_ 3.35 and δ_B_ 3.99 ppm, ^2^*J*_HH_ 13.1 Hz), and the two methyl groups give two singlet resonances (δ_N(C*H*3)2_ 2.23 and 2.45 ppm; δ_N(*C*H3)2_ 44.7 and 46.1 ppm) both in the ^1^H and the ^13^C NMR spectra (see the Supplementary Material, Figure S6). This behaviour, namely a N→Sn interaction, might be expected in solution for all the heteroleptic compounds described in this study, but it could not be observed at the room temperature NMR time scale due to a very fast process involving de-coordination, inversion at the nitrogen atom, and re-coordination [55].

Valuable data about the coordination number at the tin atom in solution is available for the ^119^Sn NMR spectra as well, as reported previously by Holeček [50] and Otera [56]. Based on the ^119^Sn NMR resonances observed in CDCl_3_ solutions, we can assign a monomeric structure for compounds **4**–**7** with pentacoordinate Sn atoms. For the tin complexes, the chemical shifts in the ^119^Sn NMR resonances are close to those observed for the starting diorganotin(IV) dichlorides, for which a structure with pentacoordinate tin was assigned previously [37,50,51,52,53,54]. Moreover, the ^1^H and the ^13^C NMR spectra of compound **7** at room temperature clearly show the intramolecular coordination of the nitrogen atom in the CH_2_N(CH_3_)_2_ pendant arm to tin. The ^119^Sn NMR resonance for **7** (δ = −104.6 ppm) has a very similar value with those observed for compounds **5** and **6**, and close to that observed for compound **4**. Anyway, the ^119^Sn chemical shifts for our compounds fall in the range reported by Otera for pentacoordinate diorganotin(IV) species (−90 ÷ −330 ppm). In this way, we can assume the existence of pentacoordinated tin atoms in solution, with *C*,*N*-chelating ligands, similarly with the solid state structures determined for the decomposition products **4-a** and **5-a**.

In the case of compound **9**, the C*H*_2_N(C*H*_3_)_2_ protons give very broad ^1^H NMR resonances at room temperature, thus suggesting a dynamic behaviour in solution, as described above, but slower at room temperature than observed in compounds **4**–**6**. To further investigate this behaviour, we performed low temperature ^1^H NMR experiments (see the Supplementary Information, Figure S8), and we could observe that at low temperatures, the C*H*_2_N protons and the two N(C*H*_3_)_2_ groups, respectively, are no more equivalent (at −30 °C δ(^1^H)_Me_ 2.24 and 2.46 ppm, and δ_A_(^1^H)_CH2_ 3.47 and δ_B_(^1^H)_CH2_ 3.94 ppm). We could calculate the free enthalpy, ΔG^#^ = 58.7 KJ/mol, at the coalescence temperature T_c_ = 15 °C, for the dynamic process suffered by the C*H*_2_ protons in the CH_2_N(CH_3_)_2_ pendant arm, and a ΔG^#^ = 70.8 KJ/mol at T_c_ = 10 °C for the two methyl groups. We can conclude that for compound **9,** the variable-temperature ^1^H NMR spectra proved that under 0 °C, the nitrogen atom in the CH_2_N(CH_3_)_2_ pendant arm remains coordinated with tin, while above this temperature the compound undergoes a dynamic process. In this way, we assume that at low temperatures compound **9** has the same molecular structure as that one determined in solid state by single-crystal X-ray diffraction, as discussed below. For compound **8,** we can assume a similar behaviour, where the N→Sn coordination involving the nitrogen atom in the pendant arm is present at low temperatures as well, while at room temperature the compound exhibits fluxionality in solution, and we expect for this compound, at low temperatures, a similar structure with that determined by single-crystal X-ray diffraction. Moreover, the chemical shift values of the ^119^Sn resonances for these two compounds suggest the existence of intramolecularly coordinated pendant arms, which results in five-coordinate tin atoms [56]. The ^119^Sn chemical shift for **8** at −252.7 ppm is close to those observed previously for the related [2-(Me_2_NCH_2_)C_6_H_4_](^n^Bu)Sn(NCS)_2_ (−266.6 ppm) and [2-(Me_2_NCH_2_)C_6_H_4_](Ph)Sn(NCS)_2_ (−329.3 ppm) [44], while for **9** the ^119^Sn resonance was observed at −161.8 ppm. These values are consistent with pentacoordinate Sn compounds [56], similarly to their solid state structure.

′The ESI+ mass spectra show for all compounds of type RR′Sn(SeCH_2_CH_2_pz)_2_ the base peak for the cation [RR′Sn(SeCH_2_CH_2_pz)]^+^, formed during ionization, at the m/z values 407.02210 for cpd. **2** (calcd. 407.01935 for C_13_H_25_N_2_SeSn), 446.06921 for **3** (calcd. 446.94169 for C_17_H_15_N_2_SeSn), 441.99945/441.99963 for compounds **4** and **9** (calcd. 441.99895/441.99915 for C_15_H_22_N_3_SeSn), 484.04724/484.04731 for compounds **5** and **7** (calcd. 484.04590 for C_18_H_28_N_3_SeSn), and 504.01658 for **6** (calcd. 504.01460 for C_20_H_24_N_3_SeSn), while for [2-(Me_2_NCH_2_)C_6_H_4_](Me)Sn(SCN)_2_ (**8**) was observed the base peak for the cation [M-SCN]^+^ at m/z 326.99713 (calcd. 326.99724 for C_11_H_15_N_2_SSn). For the tin(II) compound Sn(SeCH_2_CH_2_pz)_2_ (**10**), the mass spectrum showed the base peak at m/z 292.87903 (calcd. 292.87850 for C_5_H_7_N_2_SeSn).

### 2.3. X-Ray Diffraction Studies

#### 2.3.1. Crystal and Molecular Structure of {[2-(Me_2_NCH_2_)C_6_H_4_](Me)SnSe}_2_ (**4**-**a**) and {[2-(Me_2_NCH_2_)C_6_H_4_](^n^Bu)SnSe}_2_ (**5-a**)

**Figure 2:**
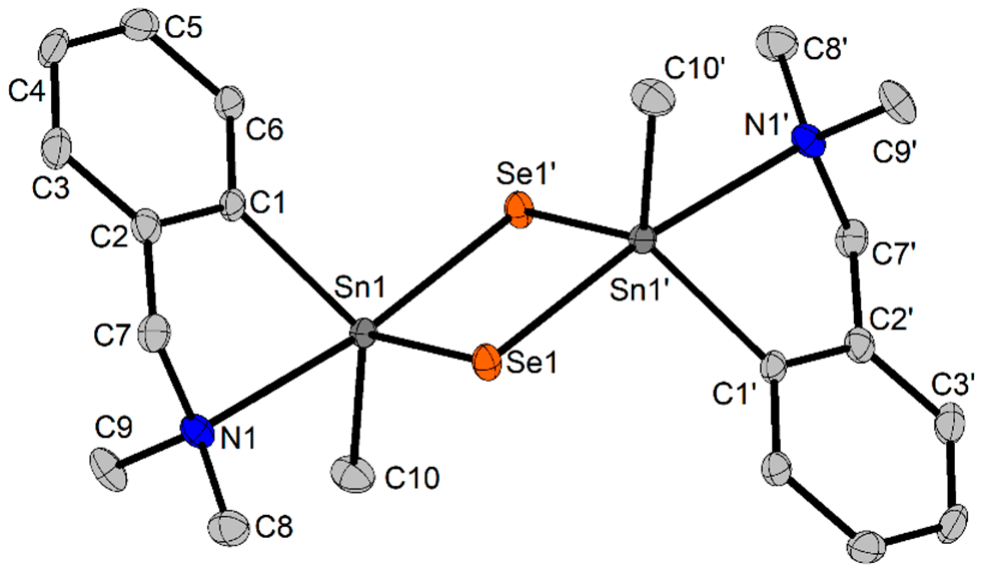
Thermal ellipsoid representation of a dimeric unit in compound **4-a**. Hydrogen atoms are omitted for clarity. Symmetry equivalent positions (-x,1-y,-z) are given by “prime”.

Our attempts to grow single-crystals of compound **4** from a mixture of CH_2_Cl_2_ and n-hexane resulted in a decomposition solid product with a dimeric structure, namely {[2-(Me_2_NCH_2_)C_6_H_4_](Me)SnSe}_2_ (**4-a**). Compound **5**, which was isolated as an oil, decomposed in CDCl_3_ solution in a similar way, and we observed the formation of a solid microcrystalline product in the NMR tube after several days. The X-ray diffraction studies also revealed for this compound the formation of the dimeric species {[2-(Me_2_NCH_2_)C_6_H_4_](^n^Bu)SnSe}_2_ (**5-a**). We have to mention that it was previously reported that in CHCl_3_ or CH_2_Cl_2_ solutions, the tin(IV) complex [Sn(S_2_CNEt_2_)_4_] decomposed with the formation of the dimeric [SnS(S_2_CNEt_2_)_2_]_2_ with a planar Sn_2_S_2_ core, and S(S_2_CNEt_2_)_2_ [57], in a way similar to what we found for compounds **4** and **5**. The dimeric structure of compound **4-a** is depicted in Figure 2, while that of **5-a** is given in the Supplementary Information, Figure S9. Important interatomic distances and bond angles are given in Table 3.

Several similarities in the molecular structures of these compounds can be pointed out.

The molecules are associated in dimeric units by bridging selenium atoms, which interact at very similar distances with both tin atoms (range 2.5033(3) Å–2.6545(3) Å, vs. Σr_cov_(Sn,Se) 2.59 Å and Σr_vdW_(Sn,Se) 4.24 Å) [58]. These values are suggestive of a tin–selenium primary interaction of type [RR′Sn^+^–Se^−^] for each selenium atom, with the calculated bond order for such an interaction being 2.57 Å, vs. the much shorter value (2.37 Å) calculated for an [RR′Sn=Se] structure [16]. A single Sn–Se bond can be assigned for compounds **4** and **5** in solution as well, based on the ^1^*J*_SnSe_ coupling constant, which falls in the range typical for single Sn–Se bonds reported for other tin organoselenolates [36]. In this way, planar Sn_2_Se_2_ cores are formed in each compound, with Se–Sn–Se angles of 94.73(1)°, and 94.45(1)° in **4-a**, and **5-a**, respectively, and the organic groups displayed *trans* each other, above and beneath the Sn_2_Se_2_ plane. Moreover, the tin atoms are very close each other, at Sn···Sn distances of 3.4952(4) Å, and 3.5183(4) Å in **4-a**, and **5-a**, respectively, much shorter than Σr_vdW_(Sn,Sn) of 4.84 Å [58].

The nitrogen atoms in the pendant arms are intramolecularly coordinated to tin, thus resulting in tin(IV) hypercoordinated species, namely *10-Sn-5* in **4-a** and **5-a** [59].

In both compounds, the five atoms attached to tin are displayed in a distorted trigonal bipyramidal coordination environment (τ_5_ = 0.81 [60]), with the nitrogen atom in the pendant arm and the selenium of the partner molecule in the dimer in apices (N1–Sn1–Se1′ 173.16(1)° in **4-a** and 172.01(6)° in **5-a**).

The coordination geometry of selenium is angular, with Sn–Se–Sn angles of 85.27(1)°, and 85.55(1)° in compounds **4-a** and **5-a**, respectively.

As a result of the intramolecular N→Sn coordination, a non-planar SnC_3_N five membered ring is formed, and consequently the compounds display planar chirality [61], where nitrogen is the pilot atom and the C_6_H_4_ ring is the chiral plane, thus resulting in *R_N_* and *S_N_* isomers, with respect to the two SnC_3_N five membered rings in a dimeric unit. In addition, due to the positions of the two selenium atoms, one in apices and the other in the equatorial plane, the tin atom becomes chiral itself, and in this way each dimer in the crystal displays *C/A* isomerism as well [61]. As a consequence, the crystal structures contain dimers formed by *C_Sn_R_N_*_1_ and *A_Sn_*_′_*S_N_*_1′_ isomers, both in **4-a** and **5-a**.

A close look at the crystals of these compounds revealed chain-like supramolecular associations with dimers (see the Supplementary Information, Figures S10 and S11). The chains are formed by CH···Se interactions in **4-a** (H5···Se 3.028 Å, vs. Σr_vdW_(H,Se) 3.10 Å [58]. In **5-a,** a CH_2_ proton in the ^n^Bu group is involved in inter-dimer π HCH···Cg contacts (H10B···Cg 2.81 Å, γ = 5.8°, C10–H10B···Cg 141°) [62,63].

#### 2.3.2. Crystal and Molecular Structure of [2-(Me_2_NCH_2_)C_6_H_4_](Me)Sn(NCS)_2_ (**8**) and [2-(Me_2_NCH_2_)C_6_H_4_](Me)Sn(NCS)(SeCH_2_CH_2_pz) (**9**)

**Figure 3:**
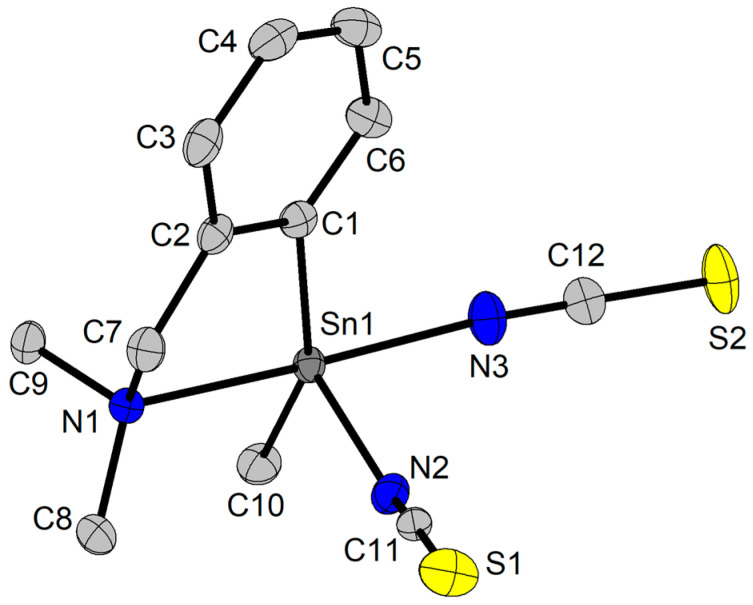
Thermal ellipsoid representation of molecule *a* in compound **8**. Hydrogen atoms are omitted for clarity.

**Figure 4:**
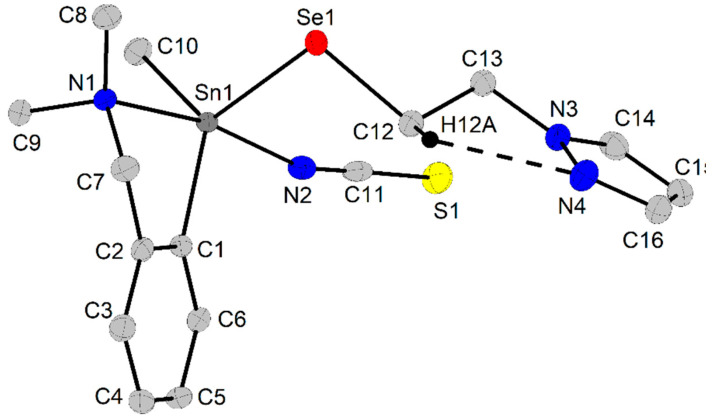
Thermal ellipsoids representation of a molecule in compound **9**. Hydrogen atoms, except that one involved in the intramolecular interaction, are omitted for clarity.

The crystal of compound **8** contains two very similar independent molecules in the unit cell. A thermal ellipsoid representation of molecule **8a** is given in Figure 3, and that of molecule **8b** is given in the Supplementary Information, Figure S12, while the molecular structure of **9** is depicted in Figure 4. Important interatomic distances and bond angles for the two independent molecules in **8** are given in Table 4, while those for the complex **9** are given in Table 3. In both species, the 2-(Me_2_NCH_2_)C_6_H_4_ group acts as a *C*,*N*-chelating moiety, while the NCS ligands behave as isothiocyanato moieties.

In the molecule of **9**, the organoselenolato pzCH_2_CH_2_Se^−^ ligand behaves as a *κSe* monodentate moiety [61], with a Sn–Se interatomic distance of 2.5298(3) Å, which is slightly shorter than those observed in R_2_Sn(2-SeC_5_H_4_N)_2_ (R = Me, 2.615(3), 2.618(3), 2.595(3) and 2.585(3) Å in the two independent molecules; R = ^t^Bu, 2.622(2) Å) [28], but it is of the same magnitude as those found in **4-a** and **5-a**, and typical for a single Sn–Se bond. In this way, compounds **8** and **9** can be described as *10-Sn-5* hypercoordinate species. The coordination geometry about tin is a distorted square pyramide in **8a** (τ_5_ = 0.26) and **8b** (τ_5_ = 0.16), with N2 and N5, respectively, in apices, and a distorted trigonal bipyramide in **9** (τ_5_ = 0.70), with N1 and N2 in apices (N1–Sn1–N2 169.64(1)°).

The intramolecular N→Sn coordination generates NC_3_Sn five-membered rings, which are not planar, but folded about the Sn···CH_2_ imaginary axis (Sn1···C7 in **8a** and in **9**, Sn2···C19 in **8b**), and, as a consequence, this induces planar chirality, which determines a racemic mixture of *R_N_* and *S_N_* isomers in the crystal [61]. Moreover, the tin atom becomes chiral itself, and the presence of C/A enantiomers [61] should be taken into account as well. Based on these considerations, the crystal of **8** contains racemic mixtures of *A_Sn_*_1_*,R_N_*_1_/*C_Sn_*_1_*,S_N_*_1_ and *C_Sn_*_2_*,S_N_*_4_/*A_Sn_*_2_*,R_N_*_4_, while **9** contains a racemic mixture of *A_Sn_*,*S_N_*_1_ and *C_Sn_*,*R_N_*_1_ isomers. The isothiocyanato ligands are essentially linear in both compounds, with N=C=S angles in the range 177.35(1)–179.58(1)°.

In the crystal of **8**, the molecules are associated in polymeric chains (see Supplementary Information, Figure S13) by NCS ligands (S3···Sn1 3.2876(6) Å vs. Σr_cov_(Sn,S) 2.44 Å and Σr_vdW_(Sn,S) 4.31 Å [58]). If we take into account this interaction as well, the coordination geometry about tin becomes distorted octahedral, and the molecules can be described as a hypercoordinate *12-Sn-6* species with bridging NCS ligands. Further, S···H contacts (S4···H21A 2.919 Å, S4···H17 2.717 Å, and S4···H7A 3.008 Å, vs. Σr_vdW_(S,H) 3.10 Å) lead to a 3D supramolecular network (see the Supplementary Information, Figure S14).

A close look at the crystal of **9** revealed π H_2_CH···Cg interactions with the participation of a proton from the methyl group attached to tin, namely H10C···Cg(C1-C6) = 3.01 Å (see Supplementary Information Figure S15).

### 2.4. Thermal Behaviour

In order to assess the potential of our compounds for the formation of tin selenides, we investigated the thermal decomposition of compound **4** (M = 616.12) both in inert atmosphere (argon) and in synthetic air. In argon atmosphere, no mass loss was observed until 200 °C, but after this, temperature decomposition occurs until 346 °C, with a mass loss of 76.48% (471 g/mol) and a residue of 145 g/mol (23.52%). The massive mass loss of 76.48% suggests the elimination of Sn(SeCH_2_CH_2_pz)_2_ (M = 467). In a second step, after a plateau, almost all the residue was volatilised above 800 °C. As the weight of the residue after the first step was significantly below the mass of the expected SnSe, we investigated the thermal behaviour of the tin(II) complex Sn(SeCH_2_CH_2_pz)_2_.(cpd. **10**,) in the same conditions. In this case, we observed a mass loss of 58.65%, which might be assigned to the diorganoselenide Se(CH_2_CH_2_pz)_2_, and a final residue corresponding to SnSe (m = 193 g/mol, 41.35%), which is close to the calculated SnSe content (m = 196, 41.97%) in the precursor. The low weight of the residue remaining from compound **4** might be explained by a partial sublimation of the tin compound before decomposition, taking into account that most of the organotin complexes are volatile species [32].

Further thermogravimetric analyses were performed in air for both compounds, and almost the same behaviour was observed in the range of 200–400 °C, as described above for the experiments under argon, but the weight of the final residue corresponded to metallic tin; namely, for compound **4,** the remaining residue of 19.07% corresponded to a mass of 117 g/mol. Apparently, the decomposition proceeds in a similar way both under argon and in air, but we have to consider a more complex pathway under an air flow, based on the formation of oxidized species in the presence of oxygen, as long as both tin and selenium are prone to oxidation.

The thermal behaviour of compounds **8** and **9** was investigated in synthetic air as well, and the final residue corresponded, in both cases, to metallic tin. For compound **9** (M = 500.11), in a first degradation step, most of the compound was eliminated, and a mass loss of 70.74% (m = 355.11) could be assigned formally to Sn(SeCH_2_CH_2_pz)(SCN) (M = 350.88, calcd. 72.18%), resembling the behaviour of compound **4**. The thermograms of compounds **4**, **8**, **9**, and **10** are displayed in the Supplementary Information, Figures S16 and S17. The experimental results suggest that the investigated compounds are volatile species, which either sublime before decomposition, or decompose easily with the elimination of organoselenium species. Their high volatility recommend them as potential candidates for CVD processes. The behaviour of our compounds resembles that observed during the thermogravimetric analyses of other tin(IV) organoselenolates, i.e., R_2_Sn{SeC_5_H_3_(Me-5)N}_2_ (R = Me, Et, ^t^Bu) [29], [Sn(SePh)_2_]_n_ [64], [(PhSe)_2_Sn{N(SiMe_3_)_2_}_2_], [{(Me_3_Si)_2_N}_2_Sn(μ^2^-Se)]_2_ [65], and Me_2_Sn{2-SeC_5_H_2_(Me-4,6)_2_N}_2_ [66], where the SnSe quantity recovered as a residue from the starting precursors was lower than the theoretical content, due to a significant volatilisation before thermal decomposition.

## 3. Materials and Methods

### 3.1. General

**Scheme 2:**
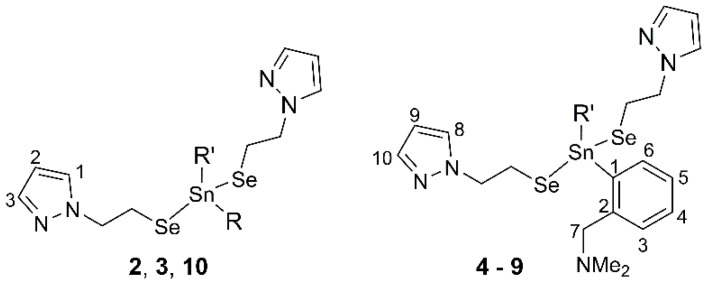
Numbering scheme for NMR assignments.

All synthetic manipulations involving air-sensitive compounds were carried out under argon atmosphere by using Schlenk techniques. Solvents were dried and distilled under argon prior to use. Starting materials such as *N,N*-dimethylbenzylamine, NaBH_4_, *^n^*BuLi (1.6 M solution in n-hexane), KSCN, Bu_2_SnCl_2_, Ph_2_SnCl_2_, and SnCl_2_ were commercial products, and they were used as received, while the diorganodiselenide (pzCH_2_CH_2_)_2_Se_2_ (**1**) [46] and the diorganotin(IV) dichlorides were prepared according to literature procedures [37,50,51,52,53,54]. Elemental analyses (CHN) were performed on a Flash EA 1112 analyser (Thermo Scientific, Waltham, MA, USA). Melting points were measured with an Electrothermal 9200 apparatus and were not corrected. The ^1^H, ^13^C{^1^H}, ^77^Se{^1^H}, and ^119^Sn{^1^H} NMR spectra, at room temperature, as well as the VT ^1^H NMR spectra for compound **9**, were recorded in CDCl_3_, on a Bruker AVANCE III instrument (Bruker, Billerica, MA, USA), operating at 400.13, 100.61, 76.31, and 149.21 MHz, respectively. The ^1^H and ^13^C{^1^H} chemical shifts are reported in *δ* units (ppm) relative to the residual peak of the solvent in the ^1^H NMR spectra (CHCl_3_, 7.26 ppm), and to the peak of the deuterated solvent (CDCl_3_, 77.16 ppm) in the ^13^C{^1^H} NMR spectra. They were assigned based on 2D correlation experiments (H,H-COSY, H,C-HSQC, and H,C-HMBC), according to the numbering shown in Scheme 2. The ^77^Se{^1^H} and ^119^Sn{^1^H} NMR resonances are reported in ppm relative to Me_2_Se and Me_4_Sn, respectively. The NMR spectra were processed using the MestReNova software (version 14) [67]. The APCI+ and ESI+ mass spectra were recorded on a Thermo Scientific LTQ-Orbitrap XL spectrometer (Waltham, MA, USA) equipped with a standard ESI/APCI source, and were processed with Thermo Xcalibur software (version 3.1) [68]. Thermogravimetric analyses (TGA) were performed in synthetic air (100 mL/min, 12 vol.% O_2_ in N_2_) or in argon (100 mL/min), using an SDT Q600 instrument (TA Instruments, USA), in the temperature range 25–1000 °C, using a heating rate of 10 °C/min.

### 3.2. Synthesis

#### 3.2.1. ^n^Bu_2_Sn(SeCH_2_CH_2_pz)_2_ (**2**)

To a yellow solution of (pzCH_2_CH_2_)_2_Se_2_ (0.131 g, 0.376 mmol) in 30 mL absolute ethanol, we added NaBH_4_ (0.035 g, 0.925 mmol, 23% excess) at ice bath temperature. A colourless solution was obtained within a few minutes. After the hydrogen release had subsided, ethanol was removed at reduced pressure, and the remaining colourless solid was washed with hexane (3 × 5 mL), dried and redissolved in ethanol. Bu_2_SnCl_2_ (0.1142 g, 0.376 mmol) was added to the ethanol solution, and the stirring continued for 1.5 h. After the evaporation of ethanol, 20 mL of dry dichloromethane was added. After filtration, the solvent was removed under vacuum, and the colourless oil was washed with hexane (3 × 5 mL) and finally dried under vacuum. Yield: 0.214 g (98%); elemental anal.: calcd. for C_18_H_32_N_4_Se_2_Sn (MW 581.11): C 37.20, H 5.55, N 9.64%; found: C 37.35, H 5.63, N 9.88%; ^1^H NMR, δ: 0.90 (t, 6H, CH_2_CH_2_CH_2_C*H*_3_, ^3^*J*_HH_ 7.3 Hz), 1.30–1.39 (m, 4H, CH_2_CH_2_C*H*_2_CH_3_), 1.43–1.53 (m, 4H, C*H*_2_CH_2_CH_2_CH_3_), 1.54–1.62 (m, 4H, CH_2_C*H*_2_CH_2_CH_3_), 3.02–3.13 (m, CH_2_C*H*_2_Se), 4.32–4.38 (m, 4H, C*H*_2_CH_2_Se), 6.23 (t, 2H, pz-*H*_2_, ^3^*J*_HH_ 1.9 Hz), 7.44 (d, 2H, pz-*H*_1_, ^3^*J*_HH_ 2.0 Hz), 7.51 (d, 2H, pz-*H*_3_, ^3^*J*_HH_ 1.5 Hz); ^13^C{^1^H} NMR, δ: 13.72 (CH_2_CH_2_CH_2_*C*H_3_), 18.55 (*C*H_2_CH_2_CH_2_CH_3_, ^1^*J*_117SnC_ 326.4, ^1^*J*_119SnC_ 341.4 Hz), 18.80 (CH_2_*C*H_2_Se, ^1^*J*_SeC_ 15.5 Hz), 26.73 (CH_2_*C*H_2_CH_2_CH_3_, ^2^*J*_117SnC_ 71.3 Hz, ^2^*J*_119SnC_ 74.4 Hz), 28.89 (CH_2_CH_2_*C*H_2_CH_3_, ^3^*J*_119SnC_ 25.4 Hz), 54.75 (*C*H_2_CH_2_Se, ^3^*J*_119SnC_ 11.1 Hz), 105.42 (pz-*C*_2_), 129.61 (pz-*C*_1_), 139.79 (pz-*C*_3_); ^77^Se{^1^H} NMR, δ: −177.7 (s, ^1^*J*_117SnSe_ 1177.1, ^1^*J*_119SnSe_ 1232.2 Hz); ^119^Sn{^1^H} NMR, δ: 79.9 (s, ^1^*J*_SeSn_ 1231.9 Hz); APCI+ MS (MeOH): m/z (%) 409.02182 (100) [M-SeCH_2_CH_2_pz]^+^ (409.01994, calcd. for C_13_H_25_N_2_SeSn), 294.88016 (52) [pzCH_2_CH_2_SeSn]^+^ (294.87909 calcd. for C_5_H_7_N_2_SeSn).

Compounds **3**–**6** were prepared similarly to compound **2**.

#### 3.2.2. Ph_2_Sn(SeCH_2_CH_2_pz)_2_ (**3**)

Ph_2_Sn(SeCH_2_CH_2_pz)_2_ (**3**) was isolated as a colourless oil from (pzCH_2_CH_2_)_2_Se_2_ (0.158 g, 0.454 mmol), NaBH_4_ (0.042 g, 1.11 mmol, 22% excess), and Ph_2_SnCl_2_ (0.156 g, 0.454 mmol). Yield: 0.189 g (67%); elemental anal., calcd. for: C_22_H_24_N_4_Se_2_Sn (MW 621.1): C 42.54, H 3.90, N 9.02%; found: C 42.97, H 4.12, N 9.21%. ^1^H NMR, δ: 3.02–3.15 (m, 4H, CH_2_C*H*_2_Se), 4.21–4.29 (m, 4H, C*H*_2_CH_2_Se), 6.17 (t, 2H, pz-*H*_2_, ^3^*J*_HH_ 2.1 Hz), 7.19 (d, 2H, pz-*H*_1_, ^3^*J*_HH_ 2.3 Hz), 7.41–7.49 (m, 8H, pz-*H*_3_ + C_6_*H*_5_-*H_m+p_*), 7.52–7.72 (m, 4H, C_6_*H*_5_-*H_o_*, ^3^*J*_SnH_ 64 Hz); ^13^C{^1^H} NMR, δ: 19.7 (CH_2_*C*H_2_Se, ^1^*J*_SeC_ 61.4 Hz), 54.39 (*C*H_2_CH_2_Se), 105.42 (pz-*C*_2_), 129.42 (C_6_H_5_-*C_m_*, ^4^*J*_SnC_ 63.7 Hz), 129.47 (pz-*C*_1_), 130.60 (C_6_H_5_-*C_p_*), 136.07 (C_6_H_5_-*C_o_*, ^2^*J*_119SnC_ 48.7 Hz), 137.01 (C_6_H_5_-*C_i_*), 139.8 (pz-*C*_3_); ^77^Se{^1^H} NMR, δ: −182.3 (s, ^1^*J*_117SnSe_ 1261.7, ^1^*J*_119SnSe_ 1321.3 Hz); ^119^Sn{^1^H} NMR, δ: −23.5 (s, ^1^*J*_SeSn_ 1320.3 Hz); APCI+ MS (MeOH): m/z (%) 448.95909 (100) [M-SeCH_2_CH_2_pz]^+^ (448.95734 calcd. for C_17_H_17_N_2_SeSn), 174.97755 (35) [pzCH_2_CH_2_Se]^+^.

#### 3.2.3. [2-(Me_2_NCH_2_)C_6_H_4_](Me)Sn(SeCH_2_CH_2_pz)_2_ (**4**)

[2-(Me_2_NCH_2_)C_6_H_4_](Me)Sn(SeCH_2_CH_2_pz)_2_ (**4**) was isolated as a colourless solid product from (pzCH_2_CH_2_)_2_Se_2_ (0.150 g, 0.430 mmol), NaBH_4_ (0.045 g, 1.2 mmol, 20% excess), and [2-(Me_2_NCH_2_)C_6_H_4_](Me)SnCl_2_ (0.146 g, 0.430 mmol). For compounds **4**–**6,** the product was extracted in toluene, instead of dichloromethane. Yield: 0.177 g (67%). M.p. 53 °C. Elemental anal., calcd. for C_20_H_29_N_5_Se_2_Sn (MW 616.12): C 38.99, H 4.74, N 11.37%; found: C 38.72, H 4.87, N 11.55%. ^1^H NMR, δ: 0.96 (s, 3H, SnC*H*_3_, ^2^*J*_117SnH_ 63.2, ^2^*J*_119SnH_ 66.5 Hz), 2.24 (s, 6H, CH_2_NC*H*_3_), 3.00–3.11 (m, 4H, CH_2_C*H*_2_Se), 3.56 (s, 2H, C_6_H_4_C*H*_2_N-*H*_7_), 4.26–4.35 (m, 4H, C*H*_2_CH_2_Se), 6.19 (t, 2H, pz-*H*_9_, ^3^*J*_HH_ 1.9 Hz), 7.10–7.17 (m, 1H, C_6_H_4_-*H*_3_), 7.31–7.35 (m, 2H, C_6_H_4_-*H*_4,5_), 7.37 (d, 2H, pz-*H*_8_, ^3^*J*_HH_ 2.1 Hz), 7.48 (d, 2H, pz-*H*_10_, ^3^*J*_HH_ 2.0 Hz), 7.75–7.98 (m, C_6_H_4_-*H_6_*, ^3^*J*_SnH_ 72.9 Hz); ^13^C{^1^H} NMR, δ: 1.58 (Sn*C*H_3_, ^1^*J*_117SnC_ 479.6, ^1^*J*_119SnC_ 501.5 Hz), 19.41 (CH_2_*C*H_2_Se, ^1^*J*_SeC_ 13.1, ^2^*J*_SnC_ 63.8 Hz), 45.1 (CH_2_N*CH*_3_), 54.8 (*C*H_2_CH_2_Se, ^2^*J*_SeC_ 11.7 Hz), 63.9 (Me_2_N*C*H_2_-*C*_7_, ^3^*J*_SnC_ 27.9 Hz), 105.26 (pz-*C*_9_), 128.11 (C_6_H_4_-*C*_3_ + C_6_H_4_-*C*_5_), 129.43 (pz-*C*_8_), 130.2 (C_6_H_4_-*C*_4_, ^4^*J*_SnC_ 14.2 Hz), 137.38 (C_6_H_4_-*C*_6_, ^2^*J*_SnC_ 55.6 Hz), 138.41 (C_6_H_4_-*C*_1_), 139.5 (pz-*C*_10_), 142.97 (C_6_H_4_-*C*_2_, ^2^*J*_SnC_ 36.9 Hz); ^77^Se{^1^H} NMR, δ: −163.6 (s, ^1^*J*_117SnSe_ 1066.3, ^1^*J*_119SnSe_ 1117.5 Hz); ^119^Sn{^1^H} NMR, δ: −74.2 (s, ^1^*J*_SnSe_ 1116.4 Hz); ESI+ MS (MeOH), m/z (%): 443.99945 (100) [M-SeCH_2_CH_2_pz]^+^ (443.99954 calcd. for C_15_H_22_N_3_SeSn).

#### 3.2.4. [2-(Me_2_NCH_2_)C_6_H_4_](^n^Bu)Sn(SeCH_2_CH_2_pz)_2_ (**5**)

[2-(Me_2_NCH_2_)C_6_H_4_](^n^Bu)Sn(SeCH_2_CH_2_pz)_2_ (**5**) was obtained from (pzCH_2_CH_2_)_2_Se_2_ (0.200 g, 0.572 mmol), NaBH_4_ (0.056 g, 1.51 mmol) and [2-(Me_2_NCH_2_)C_6_H_4_](^n^Bu)SnCl_2_ (0.436 g, 1.144 mmol). The title compound was isolated as a colourless oil after separation by column chromatography, using a mixture of ethyl acetate and dichloromethane (1:1, *v*/*v*). Yield: 0.233 g (62%). Elemental anal., calcd. for C_23_H_35_N_5_Se_2_Sn (MW 658.20): C 41.97, H 5.36, N 10.34%; found: C 42.44, H 5.62, N 10.35%. ^1^H NMR, δ: 0.92 (t, 3H, SnCH_2_CH_2_CH_2_C*H*_3_, ^3^*J*_HH_ 7.4 Hz), 1.34–1.43 (m, 2H, SnCH_2_CH_2_C*H*_2_CH_3_), 1.56–174 (m, 4H, SnC*H*_2_C*H*_2_CH_2_CH_3_), 2.26 (s, 6H, CH_2_NC*H*_3_), 3.01–3.12 (m, 4H, CH_2_C*H*_2_Se), 3.54 (2H, C_6_H_4_C*H*_2_N-*H*_7_), 4.29–4.38 (m, 4H, C*H*_2_CH_2_Se), 6.21 (t, 2H, pz-*H*_9_, ^3^*J*_HH_ 2.1 Hz), 7.13–7.17 (m, 1H, C_6_H_4_-*H*_3_), 7.30–7.37 (m, 2H, C_6_H_4_-*H*_4,5_), 7.43 (d, 2H, pz-*H*_8_, ^3^*J*_HH_ 2.2 Hz), 7.49 (d, 2H, pz-*H*_10_, ^3^*J*_HH_ 1.6 Hz), 7.86–7.91 (m, 1H, C_6_H_4_-*H*_6_, ^3^*J*_SnH_ 71.6 Hz); ^13^C{^1^H} NMR, δ: 13.8 (SnCH_2_CH_2_CH_2_*C*H_3_), 19.3 (CH_2_*C*H_2_Se, ^1^*J*_SeC_ 14.4 Hz), 21.6 (CH_2_*C*H_2_CH_2_CH_3_), 24.1 (*C*H_2_CH_2_CH_2_CH_3_), 26.7 (CH_2_CH_2_*C*H_2_CH_3_), 45.3 (CH_2_N*C*H_3_), 54.7 (*C*H_2_CH_2_Se), 64.3 (Me_2_N*C*H_2_-*C*_7_), 105.2 (pz-*C*_9_), 127.9 (C_6_H_4_-*C*_4_), 128.5 (C_6_H_4_-*C*_6_), 129.9 (C_6_H_4_-*C*_5_), 130.3 (pz-*C*_8_), 137.4 (C_6_H_4_-*C*_3_), 139.4 (pz-*C*_10_), 140.7 (C_6_H_4_-*C*_1_), 143.3 (C_6_H_4_-*C*_2_); ^77^Se{^1^H} NMR, δ: −183.2 (s, ^1^*J*_117SnSe_ 1317.4, ^1^*J*_119SnSe_ 1375.5 Hz); ^119^Sn{^1^H} NMR, δ: −52.2 (s, ^1^*J*_SnSe_ 1145.2 Hz); ESI+ MS (MeOH), m/z (%): 486.04759 (100) [M-SeCH_2_CH_2_pz]^+^ (486.04649 calcd. for C_18_H_25_N_3_SeSn).

#### 3.2.5. [2-(Me_2_NCH_2_)C_6_H_4_](Ph)Sn(SeCH_2_CH_2_pz)_2_ (**6**)

[2-(Me_2_NCH_2_)C_6_H_4_](Ph)Sn(SeCH_2_CH_2_pz)_2_ (**6**) was isolated as a yellow oil from (pzCH_2_CH_2_)_2_Se_2_ (0.202 g, 0.580 mmol), NaBH_4_ (0.053 g, 1.43 mmol), and [2-(Me_2_NCH_2_)C_6_H_4_](Ph)SnCl_2_ (0.232 g, 0.580 mmol). Yield: 0.263 g (67%); elemental anal., calcd. for C_25_H_31_N_5_Se_2_Sn (MW 678.19): C 44.28, H 4.61, N 10.33%; found: C 44.72, H 4.62, N 10.44%. ^1^H NMR, δ: 1.88 (s, 6H, CH_2_NC*H*_3_), 2.87–3.03 (m, 4H, CH_2_C*H*_2_Se), 3.49 (s, 2H, C_6_H_4_C*H*_2_N-*H*_7_), 4.05–4.18 (m, 4H, C*H*_2_CH_2_Se), 6.12 (t, 2H, pz-*H*_9_, ^3^*J*_HH_ 2.02 Hz), 7.14 (d, pz-*H*_8_, ^3^*J_HH_* 2.0 Hz), 7.14–7.17 (m, 1H, C_6_H_4_-*H*_3_), 7.35–7.44 (m, 5H, C_6_H_5_-*H*_m+p_ + C_6_H_4_-*H*_4_), 7.42 (d, 2H, pz-*H*_10_, ^3^*J*_HH_ 1.8 Hz), 7.52–7.73 (m, 2H, C_6_H_5_-*H*_o_, ^2^*J*_SnH_ 71.3 Hz), 7.92–8.11 (m, 1H, C_6_H_4_-*H*_6_, ^2^*J*_SnH_ 76.9 Hz); ^13^C{^1^H} NMR, δ: 19.6 (CH_2_*C*H_2_Se, ^1^*J*_SeC_ 12.5 Hz), 45.2 (CH_2_N*C*H_3_), 54.6 (*C*H_2_CH_2_Se), 63.7 (Me_2_N*C*H_2_-*C*_7_), 105.1 (pz-*C*_9_), 128.19 (C_6_H_4_-*C*_3_), 128.27 (C_6_H_4_-*C*_5_), 128.88 (C_6_H_5_-*C*_m_), 129.04 (C_6_H_5_-*C*_p_), 129.07 (pz-*C*_8_), 129.16 (C_6_H_4_-*C*_4_), 129.78 (pz-*C*_10_) 130.3 (C_6_H_4_-*C*_p_), 134.7 (C_6_H_5_-*C*_o_), 137.7 (C_6_H_4_-*C*_6_), 139.9 (C_6_H_4_-*C*_1_), 142.9 (C_6_H_4_-*C*_2_); ^77^Se{^1^H} NMR, δ: −182.4 (s, ^1^*J*_117SnSe_ 1121.7, ^1^*J*_119SnSe_ 1173.4 Hz; ^119^Sn{^1^H} NMR, δ: −107.2 (s, ^1^*J*_SnSe_ 1171.5 Hz); ESI+ MS (MeOH), m/z (%): 506.01622 (100) [M-SeCH_2_CH_2_pz]^+^ (506.01519 calcd. for C_20_H_24_N_3_SeSn).

#### 3.2.6. Synthesis of [2-(Me_2_NCH_2_)C_6_H_4_](^n^Bu)SnCl(SeCH_2_CH_2_pz) (**7**)

To a yellow solution of (pzCH_2_CH_2_)_2_Se_2_ (0.200 g, 0.572 mmol) in 30 mL absolute ethanol, we added NaBH_4_ (0.047 g, 1.27 mmol) at ice bath temperature. A colourless solution was obtained within a few minutes. After the hydrogen release had subsided, ethanol was removed at reduced pressure, and then the remaining colourless solid was washed with hexane (3 × 5 mL), dried, and redissolved in ethanol. The obtained solution was added dropwise to a solution of [2-(Me_2_NCH_2_)C_6_H_4_](^n^Bu)SnCl_2_ (0.436 g, 1.145 mmol) in ethanol (15 mL), and the stirring continued for 2 h. After the evaporation of ethanol, 20 mL of dry toluene was added. After removing the resulting solid by filtration, the solvent was removed under vacuum, and the remaining yellow oil was washed with hexane (3 × 5 mL) and dried at reduced pressure. Yield: 0.233 g (54%). Elemental anal., calcd. for C_18_H_28_ClN_3_SeSn (MW 519.56): C 41.61, H 5.43, N 8.09%; found: C 42.37, H 5.78, N 8.24%. ^1^H NMR, δ: 0.93 (t, 3H, SnCH_2_CH_2_CH_2_C*H*_3_, ^3^*J*_HH_ 7.2 Hz), 1.37–1.50 (m, 2H, SnCH_2_CH_2_C*H*_2_CH_3_), 1.58–176 (m, 2H, SnCH_2_C*H*_2_CH_2_CH_3_), 1.80–1.90 (m, 2H, C*H*_2_CH_2_CH_2_CH_3_), 2.23 (s, 3H, CH_2_NC*H*_3_), 2.45 (s, 3H, CH_2_NC*H*_3_), 3.08–3.27 (m, 2H, CH_2_C*H*_2_Se), AB spin system, with δ_A_ 3.35 and δ_B_ 3.99 (2H, C_6_H_4_C*H*_2_N-*H*_7_, ^2^*J*_HH_ 13.1 Hz), 4.19–4.33 (m, 2H, C*H*_2_CH_2_Se), 6.15 (t, 2H, pz-*H*_9_, ^3^*J*_HH_ 2.1 Hz), 7.12–7.22 (m, 1H, C_6_H_4_-*H*_3_), 7.25 (d, 2H, pz-*H*_8_, ^3^*J*_HH_ 2.2 Hz), 7.35–7.44 (m, 2H, C_6_H_4_-*H*_4,5_), 7.45 (d, 2H, pz-*H*_10_, ^3^*J*_HH_ 1.6 Hz), 8.11–8.34 (m, 1H, C_6_H_4_-*H*_6_, ^3^*J*_SnH_ 74.3 Hz); ^13^C{^1^H} NMR, δ: 13.8 (SnCH_2_CH_2_CH_2_*C*H_3_), 20.1 (CH_2_*C*H_2_Se, ^1^*J*_SeC_ 17.4 Hz), 24.14 (CH_2_CH_2_*C*H_2_CH_3_), 26.6 (CH_2_*C*H_2_CH_2_CH_3_, ^2^*J*_117SnC_ 104.1, ^2^*J*_119SnC_ 110.7 Hz), 28.35 (CH_2_*C*H_2_CH_2_CH_3_, ^3^*J*_SnC_ 36.7 Hz), 44.7 (CH_2_N*C*H_3_), 46.1 (CH_2_N*C*H_3_), 54.58 (*C*H_2_CH_2_Se, ^2^*J*_SnC_ 8.9 Hz), 64.2 (Me_2_N*C*H_2_-*C*_7_, ^3^*J*_SnC_ 30 Hz), 105.1 (pz-*C*_9_), 127.4 (C_6_H_4_-*C*_6_, ^2^*J*_SnC_ 65.2 Hz), 128.6 (C_6_H_4_-*C*_5_, ^3^*J*_SnC_ 74.5 Hz), 129.7 (pz-*C*_8_), 130.4 (C_6_H_4_-*C*_4_, ^4^*J*_SnC_ 14.0 Hz), 137.6 (C_6_H_4_-*C*_3_, ^3^*J*_SnC_ 50.6 Hz), 139.4 (pz-*C*_8_), 139.8 (C_6_H_4_-*C*_1_), 141.9 (C_6_H_4_-*C*_2_, ^2^*J*_SnC_ 43.3 Hz); ^77^Se{^1^H} NMR, δ: −160.9 (s, ^1^*J*_117SnSe_ 1317.4, ^1^*J*_119SnSe_ 1374.9 Hz); ^119^Sn{^1^H} NMR, δ: −104.6 (s, ^1^*J*_SeSn_ 1374.4 Hz). ESI+ MS (MeOH), m/z (%): 486.04744 (100) [M-SeCH_2_CH_2_pz]^+^ (486.04649 calcd. for C_18_H_25_N_3_SeSn).

#### 3.2.7. Synthesis of [2-(Me_2_NCH_2_)C_6_H_4_](Me)Sn(SCN)_2_ (**8**)

A solution of KSCN (0.486 g, 5.00 mmol) in 25 mL methanol was added to [2- (Me_2_NCH_2_)C_6_H_4_](Me)SnCl_2_ (0.847 g, 2.500 mmol) in dichloromethane (20 mL), at room temperature, and the reaction mixture was stirred for 3 h. The solvent was removed in vacuum, and the remaining solid was treated with CH_2_Cl_2_ (25 mL). KCl was separated by filtration, and the solvent was removed at reduced pressure. The resulting colourless solid was washed with n-hexane (3 × 5 mL). Yield: 0.816 g (85%); M.p. 150 °C; elemental anal., calcd. for C_12_H_15_N_3_S_2_Sn (MW 384.11): C, 37.52, H, 3.94, N, 10.94%, found: C, 37.77, H, 4.12, N, 11.12%. ^1^H NMR, δ: 1.12 (s, 3H, SnC*H*_3_, ^2^*J*_117SnH_ 78.7, ^2^*J*_119SnH_ 82.3 Hz), 2.47 (s, 6H, CH_2_NC*H*_3_), 3.78 (s, 2H, C_6_H_4_C*H*_2_N-*H*_7_), 7.18–7.31 (m, 1H, C_6_H_4_-*H*_3_, ^4^*J*_SnH_ 34.8 Hz), 7.43–7.54 (m, 2H, C_6_H_4_-*H*_4,5_), 7.83–8.10 (m, 1H, C_6_H_4_-*H*_6_, ^3^*J*_SnH_ 90.1 Hz); ^13^C{^1^H} NMR, δ: 2.4 (br., Sn*C*H_3_), 45.2 (CH_2_N*C*H_3_), 63.32 (Me_2_N*C*H_2_-*C*_7_, *^3^J*_SnC_ 44.7 Hz, 127.7 (C_6_H_4_-*C*_6_, ^2^*J*_SnC_ 78.8 Hz), 129.5 (C_6_H_4_-*C*_5_, ^3^*J*_SnC_ 96.2 Hz), 132.0 (C_6_H_4_-*C*_4_, ^4^*J*_SnC_ 15.7 Hz), 134.7 (br., C_6_H_4_-*C*_2_), 136.8 (C_6_H_4_-*C*_3_, ^3^*J*_SnC_ 62.4 Hz), 140.9 (C_6_H_4_-*C*_1_, ^1^*J*_SnC_ 55.2 Hz), 143.50 (br., N*C*S). ^119^Sn{^1^H} NMR, δ: −252.7 (s, br.); ESI+ MS (MeOH), m/z (%):326.99719 (100) [M-SCN]^+^ (m/z 326.99724 calcd. for C_11_H_15_N_2_SSn), 136.11211 (44) [2-(Me_2_NCH_2_)C_6_H_4_].

#### 3.2.8. Synthesis of [2-(Me_2_NCH_2_)C_6_H_4_](Me)Sn(SCN)(SeCH_2_CH_2_pz) (**9**)

To a solution of (pzCH_2_CH_2_)_2_Se_2_ (0.132 g, 0.379 mmol) in 20 mL degassed absolute ethanol, NaBH_4_ (0.032 g, 0.834 mmol) was added at ice bath temperature. Afterwards, the solvent was removed, and the remaining colourless solid was washed with n-hexane (3 × 5 mL). After redissolution in ethanol, the obtained solution was added dropwise to a solution of [2-(Me_2_NCH_2_)C_6_H_4_](Me)Sn(SCN)_2_ (0.291 g, 0.758 mmol) in ethanol (10 mL), and the stirring continued overnight. The next day, ethanol was removed at reduced pressure, and 20 mL of dry toluene was added. The solid residue was separated by filtration, and, from the clear solution, the solvent was removed under vacuum. The obtained colourless solid product was washed with n-hexane (3 × 5 mL). Yield: 0.265 g (70%); M.p. 75 °C; elemental anal., calcd. for C_16_H_22_N_4_SSeSn (MW 500.11): C, 38.43, H, 4.43, N, 11.20%, found: C, 38.22, H, 4.14, N, 11.38%. ^1^H NMR (20 °C), δ: 1.01 (s, 3H, SnC*H*_3_, ^2^*J*_117SnH_ 71.3, *^2^J*_119SnH_ 74.6 Hz), 2.34 (s, br., 6H, CH_2_NC*H*_3_), 3.06–3.25 (m, 2H, CH_2_C*H*_2_Se), 3.66 (v.br., 2H, C_6_H_4_C*H*_2_N-*H*_7_), 4.26–4.34 (m, 2H, C*H*_2_CH_2_Se), 6.18 (t, 2H, pz-*H*_9_, ^3^*J*_HH_ 2.0 Hz), 7.12–7.25 (m, 1H, C_6_H_4_-*H*_3_), 7.41 (d, 1H, pz-*H*_8_, ^3^*J*_HH_ 2.1 Hz), 7.40–7.48 (m, 2H, C_6_H_4_-*H*_4,5_), 7.46 (d, 1H, pz-*H*_10_, ^3^*J*_HH_ 1.8 Hz), 7.9–8.1 (m, 1H, C_6_H_4_-*H*_6_, ^3^*J*_SnH_ 78.1 Hz). ^1^H NMR (−50 °C), δ: 1.04 (s, 3H, SnC*H*_3_, ^2^*J*_117/119SnH_ 74.3 Hz), 2.26 (s, 3H, CH_2_NC*H*_3_), 2.49 (s, 3H, CH_2_NC*H*_3_), 3.01–3.20 (m, 2H, CH_2_C*H*_2_Se), AB spin system with δ_A_ 3.47 and δ_B_ 3.94 (2H, C_6_H_4_C*H*_2_N-*H*_7_, ^2^*J*_HH_ 14.1 Hz), 4.28–4.38 (m, 2H, C*H*_2_CH_2_Se), 6.21 (br., 1H, pz-*H*_9_), 7.18–7.27 (m, 1H, C_6_H_4_-*H*_3_), 7.33 (d, 1H, pz-*H*_8_, ^3^*J*_HH_ 2.1 Hz), 7.44–7.50 (m, 2H, C_6_H_4_-*H*_4,5_), 7.51 (br., 1H, pz-*H*_10_), 7.9–8.1 (m, 1H, C_6_H_4_-*H*_6_, ^3^*J*_SnH_ 76.5 Hz).^13^C{^1^H} NMR (20 °C), δ: 1.42 (Sn*C*H_3_, ^1^*J*_117SnC_ 568.3, ^1^*J*_119SnC_ 595.3 Hz), 19.9 (CH_2_*C*H_2_Se, ^1^*J*_SeC_ 20.7 Hz), 45.2 (br., CH_2_N*C*H_3_), 54.3 (*C*H_2_CH_2_Se, ^2^*J*_SeC_ 13.6 Hz), 63.8 (Me_2_N*C*H_2_-*C*_7_, ^3^*J*_SnC_ 38.6 Hz), 105.4 (pz-*C*_9_), 127.44 (C_6_H_4_-*C*_3_, ^3^*J*_SnC_ 69.2 Hz), 129.1 (C_6_H_4_-*C*_5_), 130.1 (pz-*C*_8_), 131.0 (C_6_H_4_-*C*_4_, ^4^*J*_SnC_ 14.7 Hz), 136.9 (C_6_H_4_-*C*_6_, ^2^*J*_SnC_ 47.7 Hz), 137.0 (C_6_H_4_-*C*_1_), 139.6 (pz-*C*_10_), 141.4 (C_6_H_4_-*C*_2_). The resonance for (S*C*N) was not observed; ^77^Se{^1^H} NMR (20 °C), δ: −167.1 (s, ^1^*J*_117SnSe_ 1353.7, *^1^J*_119SnSe_ 1422.3 Hz); ^119^Sn{^1^H} NMR (20 °C), δ: −161.8 (br., ^1^*J*_SeSn_ 1427.1); ESI+ MS (MeOH): m/z (%) 443.99915 (100) [M-SCN]^+^ (443.99954 calcd. for C_15_H_20_N_3_SeSn).

#### 3.2.9. Synthesis of Sn(SeCH_2_CH_2_pz)_2_ (**10**)

To a solution of (pzCH_2_CH_2_)_2_Se_2_ (0.302 g, 0.867 mmol) in absolute ethanol, NaBH_4_ (0.078 g, 2.11 mmol) was added. Subsequently, SnCl_2_ (0.164 g, 0.867 mmol) was added. The mixture was stirred for 2 h under argon. Then, EtOH was removed under vacuum, and the compound was redissolved in toluene. The remaining solid residue was separated by filtration, and, from the clear solution, the solvent was removed at reduced pressure. The title compound resulted in a yellow solid. Yield: 0.169 g (42%); M.p. 195 °C. Elemental anal., calcd. for C_10_H_14_N_4_Se_2_Sn (MW 466.90): C, 25.73, H, 3.02, N, 12.00%, found: C, 25.54, H, 3.48, N, 11.89%. ^1^H NMR, δ: 3.18–3.39 (m, 4H, CH_2_C*H*_2_Se), 4.39–4.52 (m, 4H, C*H*_2_CH_2_Se), 6.23 (t, 2H, pz-*H*_2_, ^3^*J*_HH_ 2.1 Hz), 7.43 (d, 2H, pz-*H*_1_, ^3^*J*_HH_ 2.1 Hz), 7.53 (d, 2H, pz-*H*_3_, ^3^*J*_HH_ 1.6 Hz); ^13^C{^1^H} NMR, δ: 23.0 (CH_2_*C*H_2_Se, ^1^*J*_SeC_ 19.5 Hz), 54.0 (*C*H_2_CH_2_Se, ^2^*J*_SeC_ 16 Hz), 105.7 (pz-*C*_2_), 129.9 (pz-*C*_1_), 140.1 (pz-*C*_3_); ^77^Se{^1^H} NMR, δ: −11.5 (s, ^1^*J*_117SnSe_ 1447.1, ^1^*J*_119SnSe_ 1513.5 Hz); ^119^Sn{^1^H} NMR, δ: −167.6 (s, ^1^*J*_SeSn_ 1511.4 Hz); ESI+ MS (MeOH), m/z (%):293.03705 (100) [M-SeCH_2_CH_2_pz]^+^ (293.87127 calcd. for C_5_H_7_N_2_SeSn), 174.97684 (38) [SeCH_2_CH_2_pz]^+^.

### 3.3. Crystal Structure Determination

Single crystals suitable for X-ray diffraction were obtained by slow diffusion from a mixture of solvents, namely CH_2_Cl_2_/n-hexane (1:3 *ν*/*ν*), for {[2-(Me_2_NCH_2_)C_6_H_4_](Me)SnSe}_2_ (**4-a**), [2-(Me_2_NCH_2_)C_6_H_4_](*^n^*Bu)Sn(NCS)_2_ (**8**), and [2-(Me_2_NCH_2_)C_6_H_4_]_2_(Me)Sn(NCS)(SeCH_2_CH_2_pz) (**9**), while single crystals of {[2-(Me_2_NCH_2_)C_6_H_4_](^n^Bu)SnSe}_2_ (**5-a**) were formed in the NMR tube from a CDCl_3_ solution of **5**. Details of the crystal structure determination and refinement are given in the Supporting Information (SI), Tables S1 and S2. The data were collected on a Bruker D–8 Venture diffractometer, using graphite-monochromated Mo-Kα radiation (λ = 0.71073 Å) from a IµS 3.0 microfocus source with multilayer optics, at 100 K. The structures were refined with anisotropic thermal parameters for non-H atoms. Hydrogen atoms were placed in fixed, idealized positions and refined with a riding model and a mutual isotropic thermal parameter. For structure solving and refinement, the Bruker APEX3 Software Package was used [69]. Intermolecular secondary bonding interactions were found with PLATON [62,63]. The drawings were created with the Diamond programme [70]. CCDC 2429261, 2429262, 2429264, and 2429265 contain the supplementary crystallographic data for compounds **4-a**, **5-a**, **8,** and **9**, respectively. The data can be obtained free of charge from The Cambridge Crystallographic Data Centre via www.ccdc.cam.ac.uk/data_request/cif (accessed on 12 March 2025).

## 4. Conclusions

The diorganotin(IV) bis(organoselenolates) RR′Sn(SeCH_2_CH_2_pz)_2_ [R = R′ = ^n^Bu (**2**), Ph (**3**); R = 2-(Me_2_NCH_2_)C_6_H_4_, R′ = Me (**4**), ^n^Bu (**5**), Ph (**6**)], [2-(Me_2_NCH_2_)C_6_H_4_](^n^Bu)SnCl(CH_2_CH_2_Se) (**7**), [2-(Me_2_NCH_2_)C_6_H_4_](Me)Sn(NCS)_2_ (**8**), and [2-(Me_2_NCH_2_)C_6_H_4_](Me)Sn(NCS)(CH_2_CH_2_Se) (**9**), as well as the tin(II) complex Sn(SeCH_2_CH_2_pz)_2_ (**10**), were prepared and structurally characterized in solution and in solid state. The ^119^Sn and the ^77^Se NMR spectra showed only one resonance for each compound, accompanied by ^1^*J*_SeSn_ and ^1^*J*_SnSe_ coupling constants, thus suggesting that the organoselenolato ligands behave as monodentate *κSe* moieties. The ^1^H and the ^13^C NMR spectra gave no clear evidence of *C*,*N*-coordination behaviour of the 2-(Me_2_NCH_2_)C_6_H_4_ group at room temperature, except for compound **7**, while a VT ^1^H NMR experiment for compound **9** revealed intramolecularly coordinated Me_2_NCH_2_ pendant arms at low temperatures. The ESI+/APCI+ HRMS spectra showed the base peak for the cations [RR′Sn(SeCH_2_CH_2_pz)]^+^ resulting from ionization.

Single-crystal X-ray diffraction studies evidenced the *C*,*N*-chelating behaviour of the 2-(Me_2_NCH_2_)C_6_H_4_ group in the dimeric species **4-a** and **5-a**, resulted from the formal elimination of Se(CH_2_CH_2_pz)_2_ among each two molecules of **4** and **5**, respectively. The *C*,*N*-chelating behaviour of the 2-(Me_2_NCH_2_)C_6_H_4_ group was observed in the solid state structures of **8** and **9** as well.

Based on the ^1^*J*_SnC_ coupling constant in the ^13^C NMR spectrum of **2**, we could assign a distorted tetrahedral coordination geometry about the tin atom in solution, and we can assume a similar coordination geometry for compound **3**, both being liquids at room temperature. For the compounds bearing a 2-(Me_2_NCH_2_)C_6_H_4_ group attached to tin, based on the NMR spectra, we assume a temperature dependent dynamic process in solution which involves decoordination, inversion at nitrogen, and re-coordination to tin. The single-crystal molecular structure of compounds **4-a**, **5-a**, **8**, and **9** are consistent with hypercoordinated *10-Sn-5* species.

Preliminary thermogravimetric experiments showed that the solid species **4**, **8**, **9**, and **10** might be considered as potential molecular precursors for tin chalcogenides, mostly in CVD processes, due to their high volatility. Further investigations are necessary to establish the thermal behaviour of these molecular species.

## Figures and Tables

**Table 1:** ^1^*J*_119SnC_ coupling constant, and calculated C–Sn–C angle in solution, for compound **2**.

Compound	^13^C{^1^H} NMR	
	^1^*J*(^119^Sn,^13^C)	C–Sn–C
**2**	341.4	106.7° ^a^ 108.8° ^b^

^a 1^*J* = 11.4θ–875; ^b 1^*J* = 9.99θ–746.

**Table 2:** ^77^Se and ^119^Sn NMR resonances for compounds **1**–**10** and the starting RR′SnCl_2_.

Cpd.	δ_77Se_ (ppm)	^1^*J*_117SnSe_/^1^*J*_119SnSe_ (Hz)	δ_119Sn_ (ppm)	^1^*J*_Se119Sn_ (Hz)	RR′SnCl_2_	δ_119Sn_ (ppm)
**1**	292.1 [25]	-	-	-	-	-
**2**	−177.7	1177.1 (^117^Sn) 1232.2 (^119^Sn)	79.9	1231.9	^n^Bu_2_SnCl_2_	126.3 [50]
**3**	−182.3	1261.7 (^117^Sn) 1321.3 (^119^Sn)	−23.5	1320.3	Ph_2_SnCl_2_	−26.7 [51]
**4**	−163.6	1066.3 (^117^Sn) 1117.5 (^119^Sn)	−74.2	1116.4	R′MeSnCl_2_	−97.3 [52,53]
**5**	−161.0	1317.4 (^117^Sn) 1375.5 (^119^Sn)	−104.8	1380.5	R′^n^BuSnCl_2_	−103 [37]
**6**	−182.4	1121.7 (^117^Sn) 1173.4 (^119^Sn)	−107.2	1171.5	R′PhSnCl_2_	−170 [54]
**7**	−160.9	1317.4 (^117^Sn) 1374.9 (^119^Sn)	−104.6	1374.4	R′^n^BuSnCl_2_	−103
**8**			−252.7		R′MeSnCl_2_	−97.3
**9**	−167.1	1353.7 (^117^Sn) 1422.3 (^119^Sn)	−161.8	1427.1	R′MeSn(SCN)_2_	−252.7
**10**	−11.5	1447.1 (^117^Sn) 1513.5 (^119^Sn)	−167.6	1511.4		

**Table 3:** Important interatomic distances and bond angles in **4-a**, **5-a**, and **9**.

Bond/Angle	4-a	5-a		9
Sn1–Se1	2.5033(3)	2.5317(2)	Sn1–Se1	2.5298(3)
Sn1–Se1′	2.6545(3)	2.6475(4)	Sn1–N1	2.4110(3)
Sn1–N1	2.5633(3)	2.6046(2)	Sn1–N2	2.2410(2)
Sn1–C1	2.1434(2)	2.1528(1)	Sn1–C1	2.1231(2)
Sn1–C10	2.1365(2)	2.1662(1)	Sn1–C10	2.1202(2)
Se1–Se1′	3.7960(4)	3.8025(5)	N2–C11	1.1354(1)
Sn1–Sn1′	3.4952(4)	3.5183(4)	S1–C11	1.6273(2)
Se1–Sn1–Se1′	94.73(1)	94.45(1)	N1–Sn1–Se1	89.85(1)
N1–Sn1–Se1	88.60(1)	87.11(1)	C1–Sn1–Se1	114.92(1)
N1–Sn1–Se1′	173.16(1)	172.01(6)	C1–Sn1–C10	127.58(1)
N1–Sn1–C1	73.83(1)	72.89(1)	C10–Sn1–Se1	116.46(1)
N1–Sn1–C10	85.76(1)	84.82(1)	N2–Sn1–Se1	93.37(1)
C1–Sn1–Se1	114.27(1)	111.92(1)	N2–Sn1–C1	92.91(1)
C1–Sn1–Se1′	99.35(1)	99.30(7)	N2–Sn1–C10	93.86(1)
C1–Sn1–C10	124.23(1)	123.22(1)	N2–Sn1–N1	169.64(1)
C10–Sn1–Se1	116.50(1)	118.42(1)	N2–C11–S1	179.09(1)
C10–Sn1–Se1′	98.05(1)	101.23(7)		
Sn1–Se1–Sn1′	85.27(1)	85.55(1)		

**Table 4:** Important interatomic distances and bond angles in **8**.

Bond/Angle	8a		8b
Sn1–N1	2.3926(1)	Sn2–N4	2.3804(1)
Sn1–N2	2.1183(1)	Sn2–N5	2.1567(1)
Sn1–N3	2.1874(1)	Sn2–N6	2.1848(1)
Sn1–C1	2.1082(1)	Sn2–C13	2.1178(1)
Sn1–C10	2.1075(1)	Sn2–C22	2.1051(1)
N2–C11	1.1460(1)	N5–C23	1.1615(1)
N3–C12	1.1605(1)	N6–C24	1.1229(1)
S1–C11	1.6094(1)	S3–C23	1.6159(1)
S2–C12	1.6241(1)	S4–C24	1.6229(1)
Sn1′–S3	3.2876(6)	Sn2–S2	3.1441(7)
N1–Sn1–N2	86.74(1)	N4–Sn2–N5	84.37(1)
N1–Sn1–N3	170.38(1)	N4–Sn2–N6	167.92(1)
N2–Sn1–N3	88.58(1)	N5–Sn2–N6	87.47(1)
N1–Sn1–C1	77.63(1)	N4–Sn2–C13	76.63(1)
N1–Sn1–C10	94.07(1)	N4–Sn2–C22	93.64(1)
C1–Sn1–C10	154.39(1)	C13–Sn2–C22	158.17(1)
N2–Sn1–C1	99.80(1)	N5–Sn2–C13	99.24(1)
N2–Sn1–C10	103.91(1)	N5–Sn2–C22	99.18(1)
N3–Sn1–C1	94.91(1)	N6–Sn2–C13	95.97(1)
N3–Sn1–C10	95.22(1)	N6–Sn2–C22	96.45(1)
N2–C11–S1	177.35(1)	N5–C23–S3	178.85(1)
N3–C12–S2	179.23(1)	N6–C24–S4	179.58(1)

## Data Availability

Original experimental data not provided in the Supplementary Information are available from the authors on request.

## Supplementary Materials

The following supporting information can be downloaded at https://www.mdpi.com/article/10.3390/molecules30071648/s1: NMR spectra (Figures S1–S8), Single-crystal X-ray structures (Figures S9–S15), Thermogravimetric analysis data (Figures S16 and S17), and Crystallographic data (Tables S1 and S2). Supplementary Material corresponding to the X-ray structure has been deposited in the CCDC as CCDC 2429261, 2429262, 2429264, and 2429265 (see Section 3.3).
