# Supplementary material for: Diorganotin(IV) Complexes of Organoselenolato Ligands with Pyrazole Moieties—Synthesis, Structure and Properties
Source: Molecules. 2025 Apr 7;30(7):1648. doi: 10.3390/molecules30071648 (PMC11990251; doi:10.3390/molecules30071648)
Supplement: Supplementary file 1 [file molecules-30-01648-s001.zip › molecules-3559989-supplementary.pdf]

Molecules

SUPPLEMENTARY INFORMATION

Diorganotin(IV) complexes of organoselenolato ligands with pyrazole moieties. Synthesis, structure and properties

Melinda Tamas,[1] Roxana A. Butuza,[1] Monica Dan,[2] Anca Silvestru[1]*

[1]*Supramolecular Organic and Organometallic Chemistry Centre (CCSOOM), Chemistry Department, Faculty of Chemistry and Chemical Engineering, "Babeş-Bolyai" University, 400028 Cluj-Napoca, Romania. E-mail: anca.silvestru@ubbcluj.ro*

[2]*National Institute for Research and Development of Isotopic and Molecular Technologies, 400293 Cluj-Napoca, Romania*

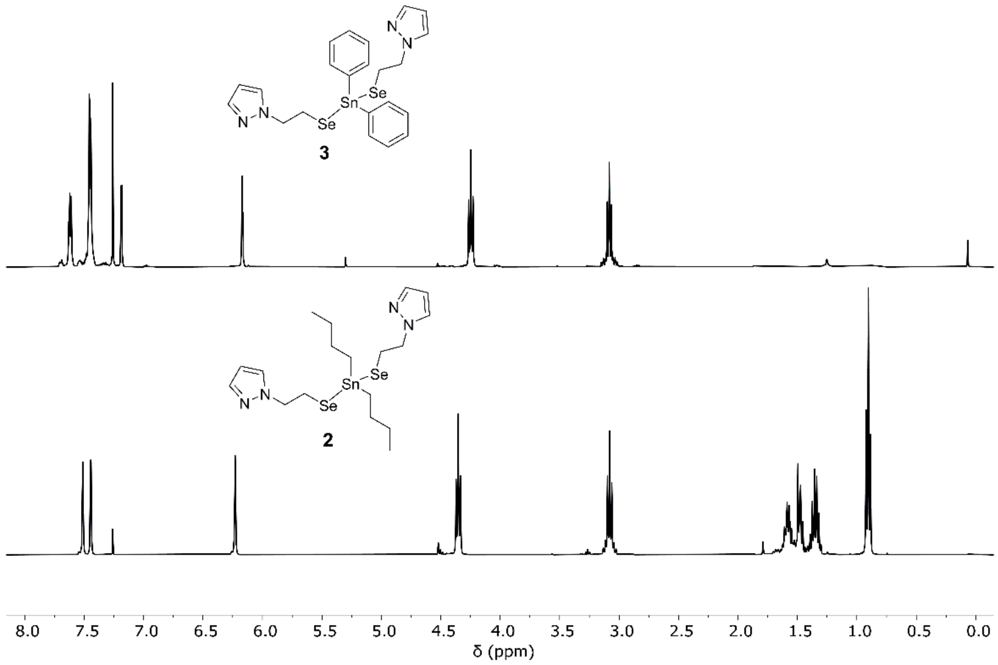

Figure S1. ^{1}H NMR spectra of compounds **2** and **3**, in $CDCl_3$

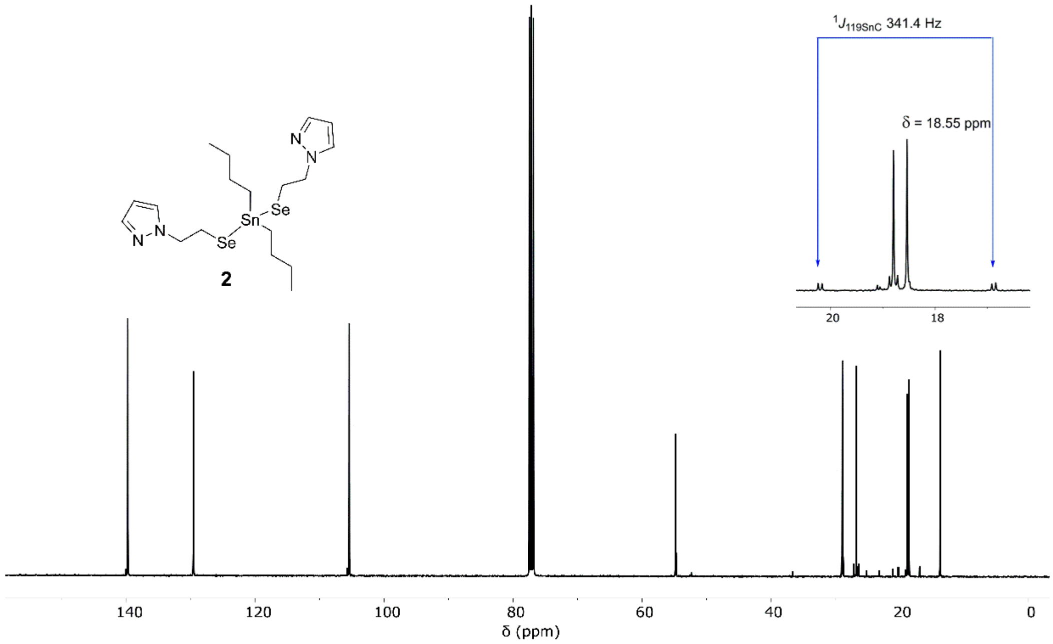

Figure S2. ^{13}C NMR spectrum of compound **2**, in $CDCl_3$

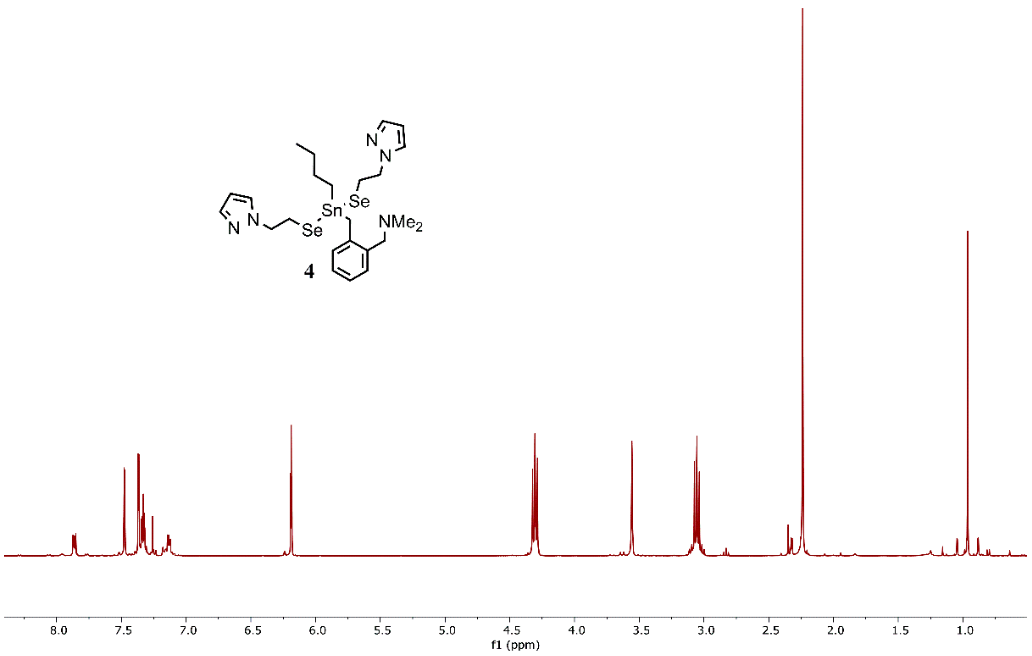

Figure S3. ^{1}H NMR spectrum of compound **4**, in $CDCl_3$

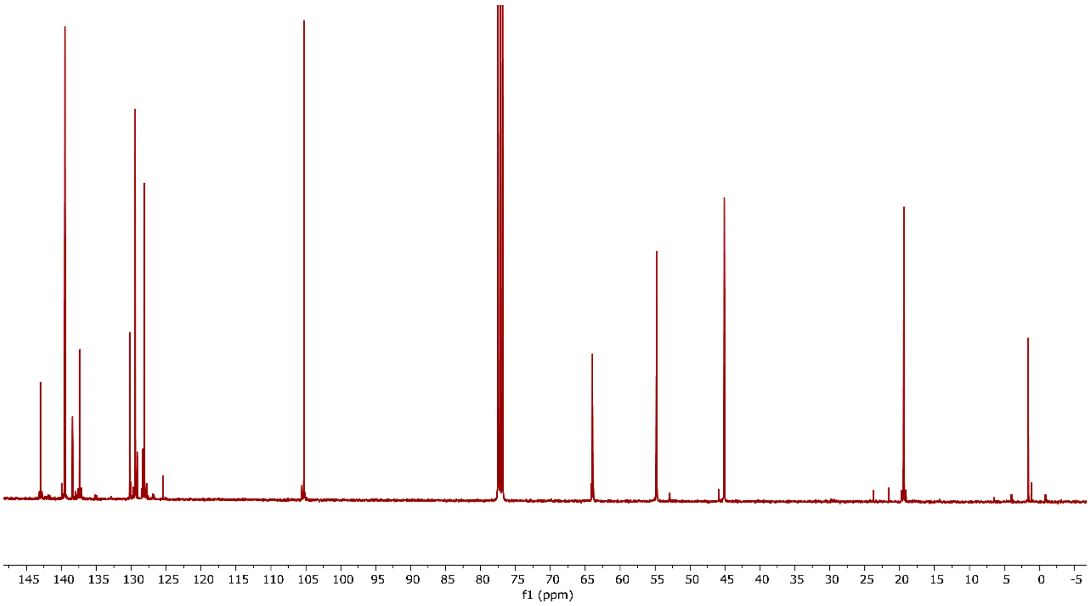

Figure S4. ^{13}C NMR spectrum of compound **4**, in $CDCl_3$

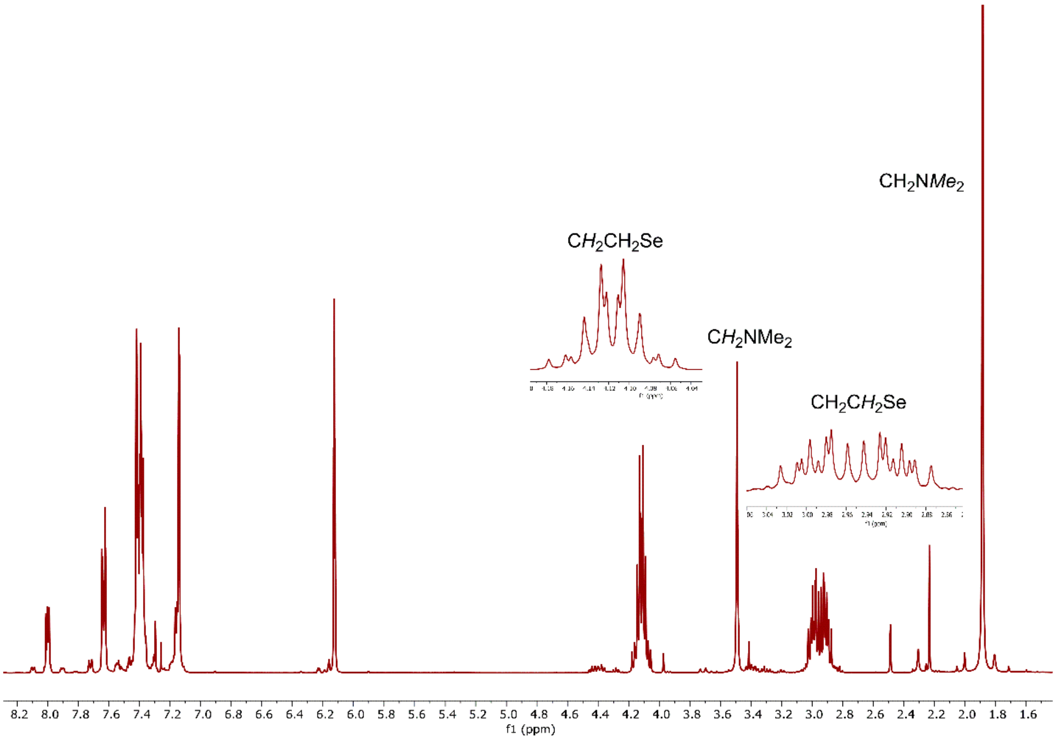

Figure S5. ^{1}H NMR spectrum of compound **6**, in $CDCl_3$

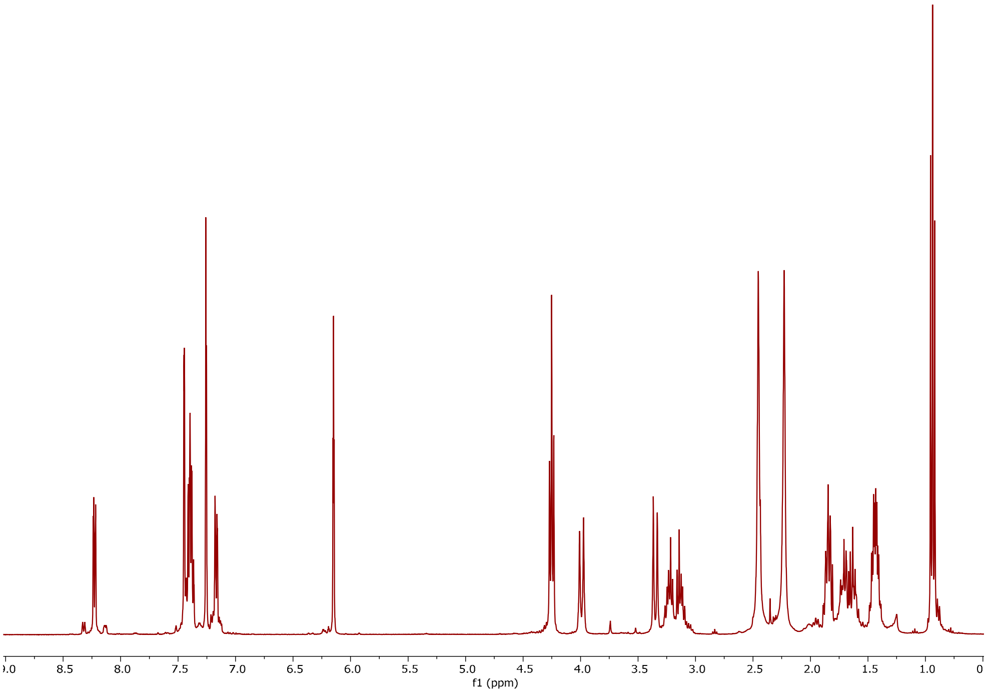

Figure S6. ^{1}H NMR spectrum of compound **7**, in $CDCl_3$

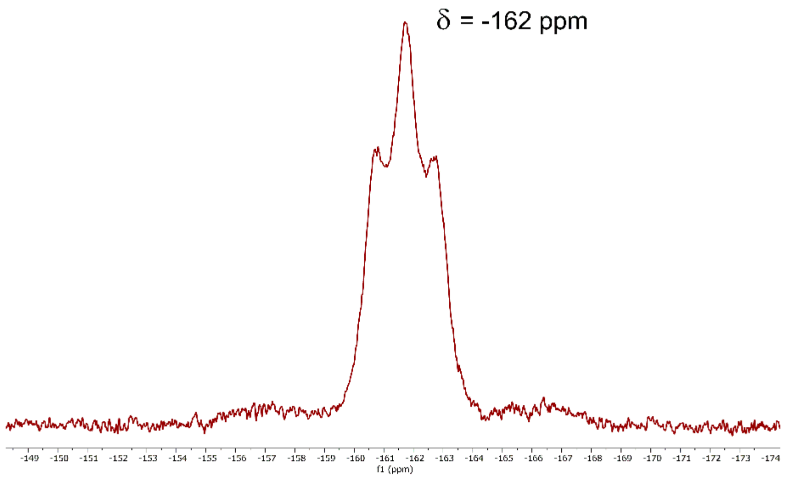

Figure S7. ^{119}Sn NMR resonance of [2-(Me_2NCH_2)C_6H_4](Me)Sn(NCS)(CH_2CH_2Se) (**9**), in $CDCl_3$

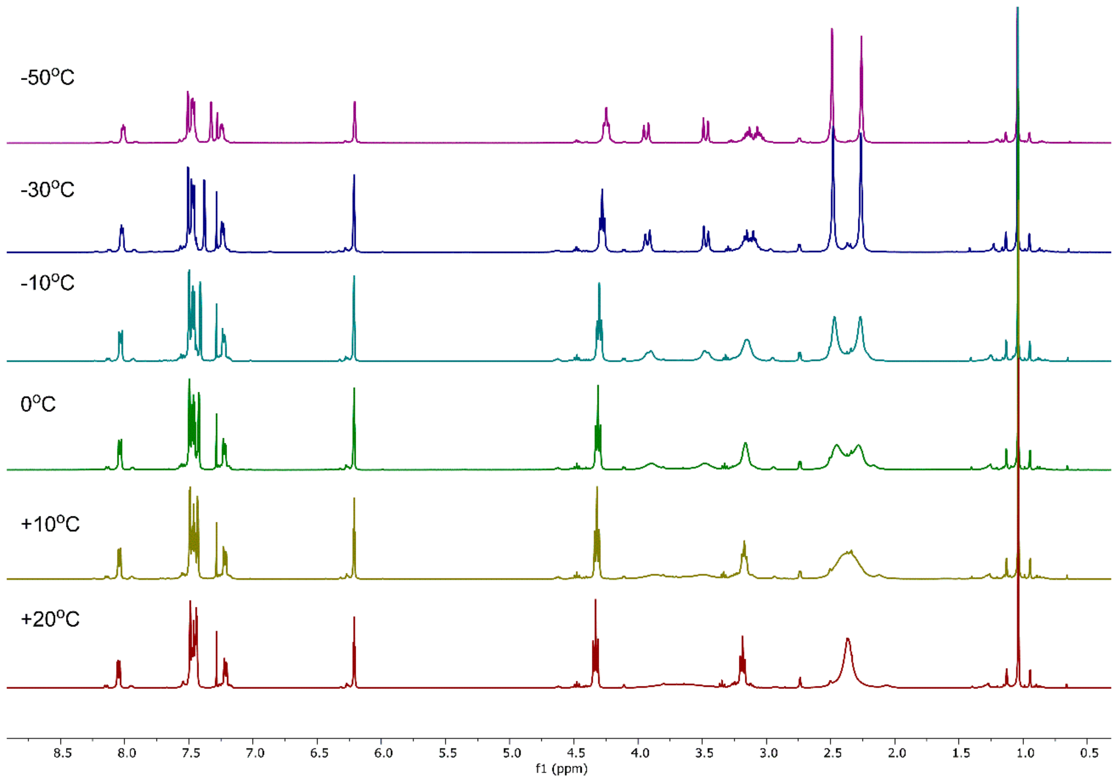

Figure S8. VT ^{1}H NMR spectra of compound **9**, in $CDCl_3$

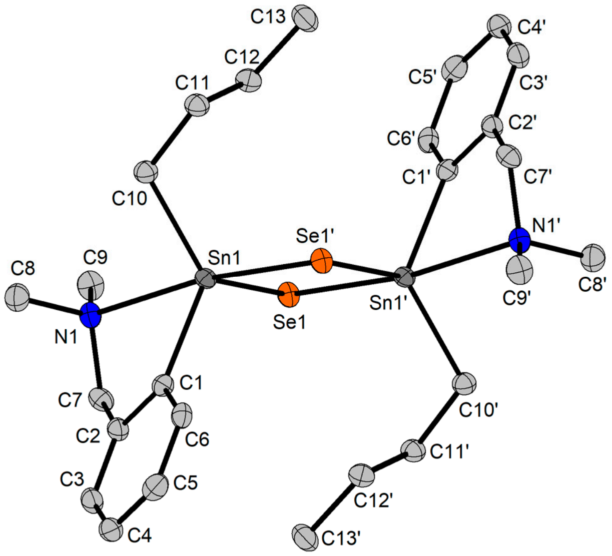

Figure S9. Thermal ellipsoids representation of a dimeric unit in compound **5-a**. Hydrogen atoms were omitted for clarity. Symmetry equivalent positions (1-x, 1-y, 1-z) are given by "prime".

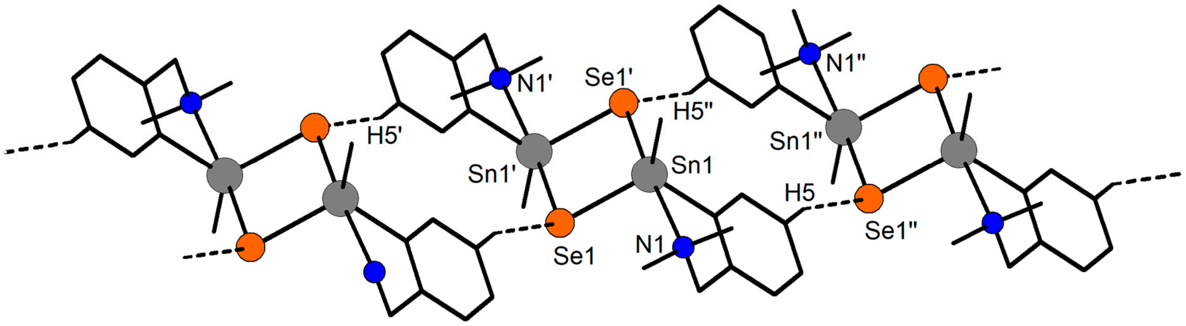

Figure S10. View along axis *c* of a chain in the crystal of **4-a**. Hydrogen atoms, except those involved in inter-dimers interactions were omitted for clarity. Symmetry equivalent positions (-x, 1-y, -z) and (1-x, 1-y, -z) are given by "prime" and "double prime", respectively.

H5"···Se1' 3.028 Å, vs. Σr_{vdW}(H,Se) 3.10 Å

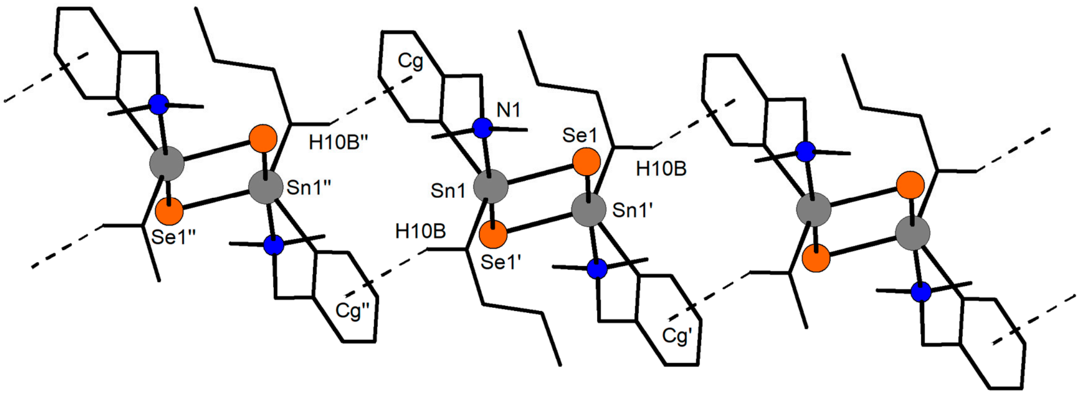

Figure S11. Best view of a chain in the crystal of **5-a**. Hydrogen atoms, except those involved in inter-dimers interactions were omitted for clarity. Symmetry equivalent positions (1-x, 1-y, 1-z) and (1-x, -y, 1-z) are given by "prime" and "double prime", respectively.

H10B"···Cg   2.81 Å

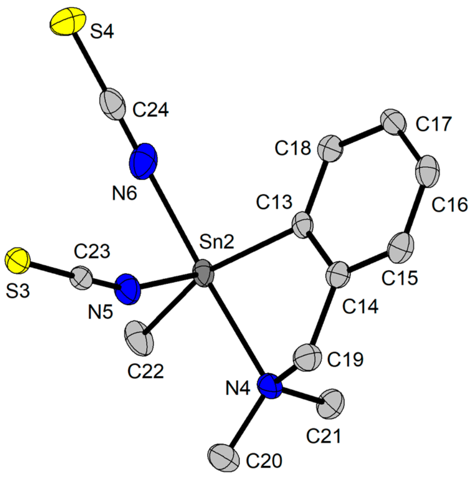

Figure S12. Thermal ellipsoids representation of molecule b in compound **8**. Hydrogen atoms were omitted for clarity.

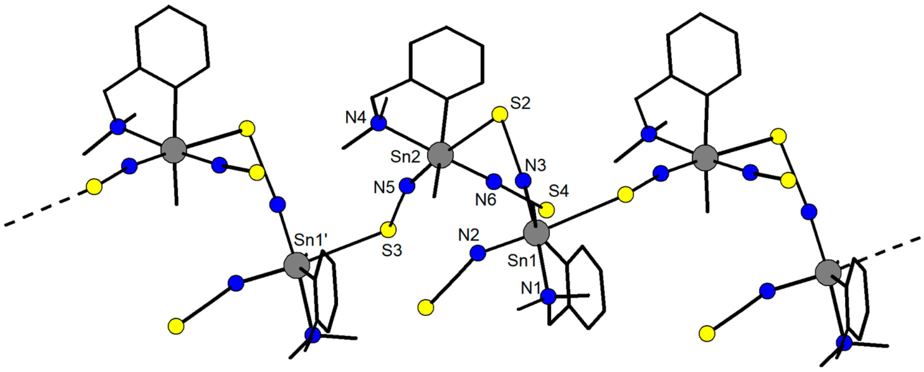

Figure S13. Best view of a chain in the crystal of **8**. Hydrogen atoms, except those involved in inter-dimers interactions were omitted for clarity. Symmetry equivalent positions (-x, 1-y, -z) are given by “prime”.

S3···Sn1'   3.2876(6) Å,   *vs*. Σr_{cov}(Sn,S) 2.44 Å and Σr_{vdW}(Sn,S) 4.31 Å

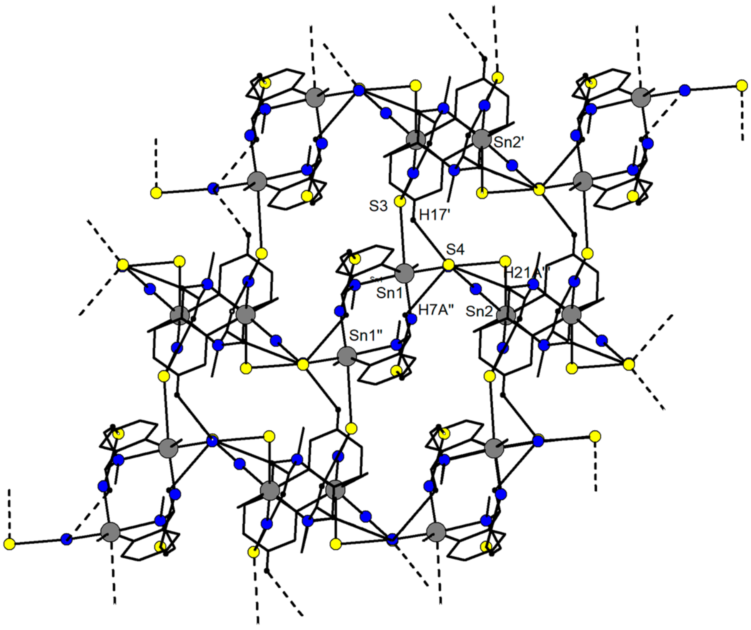

Figure S14. 3D network in the crystal of **8** (view along axis b)

Symmetry equivalent positions (1-x, 2-y, 1-z), (1/2-x, 1/2+y, 3/2-z), (3/2-x, 3/2+y, 3/2-z) are given by “prime”, “double prime”, and “triple prime”, respectively

S4···H21A'''   2.919 Å,   vs. Σr_{vdW}(S,H) 3.10 Å

S4···H17'   2.717 Å   S4···H7A''   3.008 Å

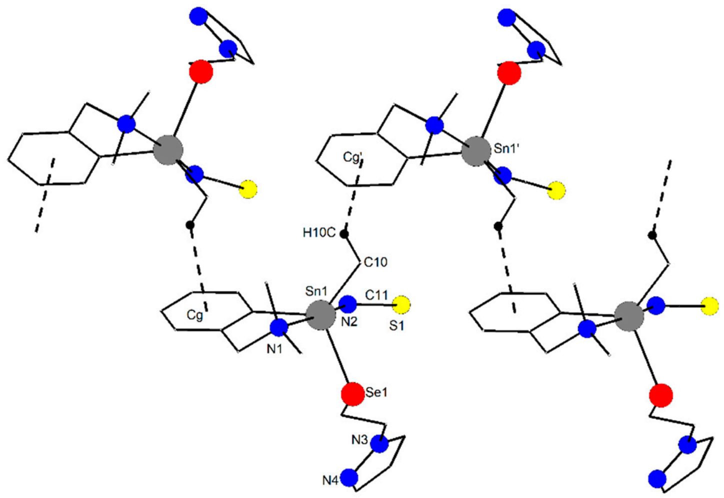

Figure S15. Best view of a chain in the crystal of **9**. Hydrogen atoms, except those involved in inter-dimers interactions were omitted for clarity. Symmetry equivalent positions (1/2+x, y, 1/2-z) are given by “prime”.

H10C···Cg' = 3.01 Å

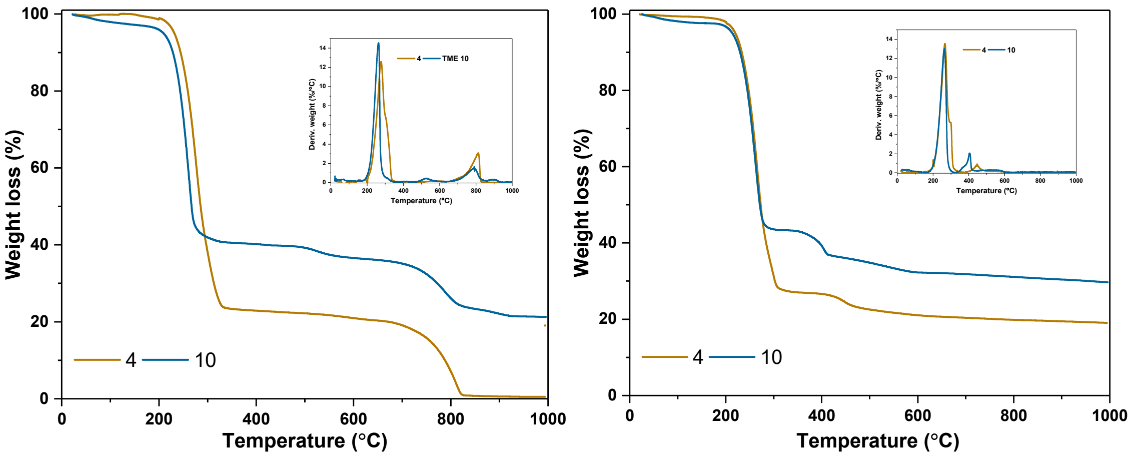

Figure S16. Thermogravimetric analysis data for compounds **4** and **10**, in argon (left), and in synthetic air (right)

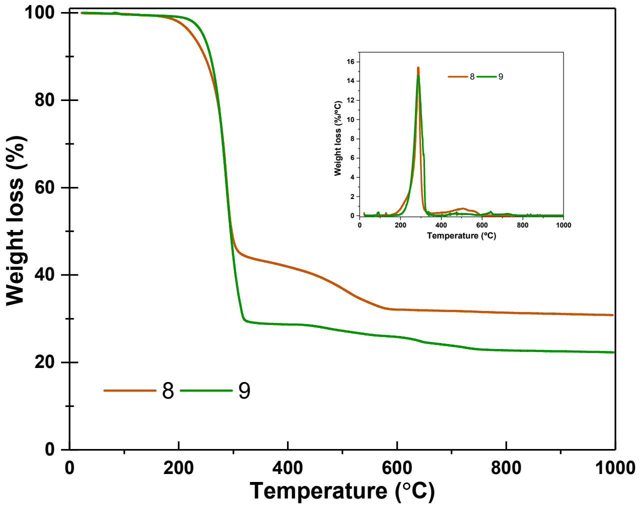

Figure S17. Thermogravimetric analysis data for compounds **8** and **9**, in synthetic air

Table S1. Crystallographic data for compounds **4-a** and **5-a**

	**4-a**	**5-a**
Empirical formula	$C_{20}H_{30}N_2Se_2Sn_2$	$C_{26}H_{42}N_2Se_2Sn_2$
Formula weight	693.76	777.91
Temperature	100.(2)	100.(2)
Wavelength	0.71073	0.71073
Crystal system	Monoclinic	Triclinic
Space group	P21/c	P-1
a [Å]	9.6290(11)	9.0921(6)
b [Å]	13.4804(14)	9.3314(6)
c [Å]	10.3069(12)	9.6736(6)
α [°]	90	115.972(2)
β [°]	117.85	93.422(2)
γ [°]	90	99.902(2)
Volume	1182.9(2)	718.36(8)
Z	2	1
Density (calculated)	1.948	1.798
Absorption coefficient	5.195	4.288
F(000)	664	380
Crystal size	0.129x0.214x0.228	0.181x 0.219x 0.223
Theta range for data collection	2.39 to 26.60	2.30 to 28.34
Index ranges	-12<=h<=12	-12<=h<=12
	-16<=k<=16	-12<=k<=12
	-12<=l<=12	-12<=l<=12
Reflections collected/unique	27714/2448	38409/3582
Completeness to theta max.	99 %	100 %
Absorption correction		
Refinement method		
Data / restrains / parameters	2448/ 0 / 121	3582 / 0 / 148
Goodness-of-fit on F^2 -S	1.297	1.044
Final R indices [I>2sigma(I)]	R1 = 0.0129	R1 = 0.0260
	wR2 = 0.0276	wR2 = 0.0492
R indices (all data)	R1 = 0.0136	R1 = 0.0368
	wR2 = 0.0361	wR2 = 0.0525
Largest diff. peak and hole	0.432 and -0.417 e/Å^3	1.065 and -0.854 e/Å^3

Table S2. Crystallographic data for compounds **8** and **9**

	**8**	**9**
Empirical formula	$C_{12}H_{15}N_3S_2Sn$	$C_{16}H_{22}N_4SSeSn$
Formula weight	384.08	500.08
Temperature	100.(2)	100.(2)
Wavelength	0.71073	0.71073
Crystal system	Monoclinic	Orthorhombic
Space group	P21/n	Pbca
a [Å]	14.7835(8)	9.1223(10)
b [Å]	12.7152(6)	15.8529(16)
c [Å]	17.2721(9)	26.319(2)
α [°]	90	90
β [°]	112.643(2)	90
γ [°]	90	90
Volume	2996.5(3)	3806.1(7)
Z	8	8
Density (calculated)	1.703	1.745
Absorption coefficient	1.970	3.369
F(000)	1520	1968
Crystal size	0.117x0.136x 0.214	0.080x0.100x0.120
Theta range for data collection	2.05 to 26.41	2.68 to 28.28
Index ranges	-18<=h<=18	-12<=h<=12
	-15<=k<=15	-21<=k<=21
	-21<=l<=21	-35<=l<=34
Reflections collected/unique	65915 / 6138	75630 / 4696
Completeness to theta max.	99.8 %	99.6 %
Absorption correction	multi-scan	
Refinement method	Full-matrix least-squares on F^2	
Data / restrains / parameters	6138 / 0 / 331	4696 / 0 / 211
Goodness-of-fit on F^2 -S	1.049	1.095
Final R indices [I>2sigma(I)]	R1 = 0.0179	R1 = 0.0173
	wR2 = 0.0394	wR2 = 0.0349
R indices (all data)	R1 = 0.0194	R1 = 0.0224
	wR2 = 0.0400	wR2 = 0.0374
Largest diff. peak and hole	1.066 and -0.662 e/Å^3	0.395 and -0.373 e/Å^3
